# BMP-2 functional polypeptides relieve osteolysis via bi-regulating bone formation and resorption coupled with macrophage polarization

**DOI:** 10.1038/s41536-023-00279-2

**Published:** 2023-02-09

**Authors:** Jiaqian Wang, Yuan Xue, Yi Wang, Chang Liu, Sihan Hu, Huan Zhao, Qiaoli Gu, Huilin Yang, Lixin Huang, Xichao Zhou, Qin Shi

**Affiliations:** 1 Department of Orthopedics, the First Affiliated Hospital of Soochow University, Medical college of Soochow University, Orthopedic Institute of Soochow University, 899 Pinghai Road, Suzhou, Jiangsu 215031 P. R. China; 2 Department of Orthopedics, Wuxi No. 5 People’s Hospital, Wuxi, Jiangsu 214000 P. R. China; 3 Department of Orthopaedic, Wuxi Ninth People’s Hospital affiliated to Soochow University, Wuxi, Jiangsu 214026 P. R. China

**Keywords:** Implants, Translational research

## Abstract

Osteolysis caused by wear debris around the prosthesis is the main reason for aseptic loosening. Extending prosthetic service life is still challenging. In this study, we first synthesized a bone morphogenetic protein-2 (BMP-2) functional polypeptide (BMP2pp), and evaluated the effects of BMP2pp on macrophage polarization and impaired osteogenesis caused by titanium (Ti) particles in vitro. Then, we delineated the impact of BMP2pp on bone formation and resorption in a mouse calvarial bone osteolysis model induced by Ti particles. The results showed that BMP2pp not only alleviated the Ti-induced inhibition of osteoblastic differentiation in human placenta-derived mesenchymal stem cells (hPMSCs) but also prevented Ti-induced M1 macrophage polarization and promoted M2 macrophage differentiation in mice. Conditioned medium from BMP2pp-activated macrophages increased the osteogenesis of hPMSCs. The western blot results indicated a significant decrease in the expression of NF-κB inducing kinase (NIK) and phospho-NF-κB p65 in bone marrow-derived macrophages treated with BMP2pp. Furthermore, we clarified the protective effect of BMP2pp on bone formation and the reduction in bone resorption coupled with the immunomodulatory properties of calvarial osteolysis in mice. In summary, BMP2pp ameliorated the Ti-mediated impairment in osteogenic potential of hPMSCs, suppressed the M1 polarization of macrophages by inhibiting the activation of the NF-κB signaling pathway, and ameliorated Ti-induced bone osteolysis. Our research suggests that BMP2pp may be a potential option for treating prosthetic loosening induced by wear debris from prostheses.

## Introduction

Artificial joint replacement is an alternative method for treating advanced arthritis. However, with the increasing number of artificial total hip and knee replacement surgeries, many patients need revision in arthroplasty because of aseptic loosening of the prosthesis^1,2^. Osteolysis caused by wear debris around the prosthesis is the main cause of aseptic loosening^3^. Wear debris from prostheses gathers between the bone-implant interface and can stimulate macrophages to produce various inflammatory mediators and cytokines. This further impairs the balance in osteoblastic bone formation and osteoclastic bone absorption, causing osteolysis around the prosthesis^4^. Although the wear rate of fourth-generation bioceramics is very low, there are still many patients who come to the hospital for treatment due to prosthesis loosening every year, and the number is increasing annually^5^. Considering the inevitability of wear debris, reducing the impact of debris may be an excellent way to prolong the life of the prosthesis^6^.

Macrophages are the first line of defense against microorganisms in the human body. These cells can phagocytize bacteria, parasites, and aging and apoptotic cells and play a crucial role in immune regulation^7^. Increasing evidence demonstrates that wear debris stimulates macrophage activation and the subsequent osteoclastic differentiation of osteoclast precursor cells, which initiates bone resorption. Indeed, recent studies showed that impaired osteogenic cell viability and/or osteoblastic differentiation caused by the stimulation of wear particles is also an important reason for osteolytic disease. Increasing evidence demonstrates that wear debris stimulates macrophage activation and the subsequent osteoclastic differentiation of osteoclast precursor cells, which initiates bone resorption. Indeed, recent studies showed that impaired osteogenic cell viability and/or osteoblastic differentiation caused by the stimulation of wear particles is also an important reason for osteolytic disease^8^. The different polarization states of macrophages and their immunoregulatory functions may play essential roles in this process^9^. Macrophages can polarize into the classically activated M1 phenotype and alternatively activated M2 phenotype depending on local conditions^10^. M1 macrophages can secrete proinflammatory factors upon stimulation, such as interleukin-1β (IL-1β), tumor necrosis factor-α (TNF-α), and interleukin-6 (IL-6). In contrast, M2 macrophages can inhibit the inflammatory response and play an important role in various types of wound repair by secreting anti-inflammatory factors and growth factors, such as interleukin-10 (IL-10), arginase-1 (Arg-1), and vascular endothelial growth factor (VEGF)^9,11^.

As one of the well-characterized transforming growth factor-β (TGF-β) superfamily factor members, bone morphogenetic protein-2 (BMP-2) exhibits excellent pro-osteogenic activity and can induce osteoblastic differentiation and enhance bone formation^12^. Recombinant human BMP-2 protein (rhBMP-2) is widely used in the clinic as a substitute for bone transplantation during surgery, such as fracture and intervertebral disc herniation, but whether it can protect against the osteolysis caused by wear debris after joint replacement is still unknown^13^. Recently, some studies have shown that the expression of BMP-2 may have a supportive effect on M2 macrophage polarization, but the specific mechanism is not clear^14^. In addition, clinical use of rhBMP-2 still has some limitations, such as high cost, difficult storage, and many side effects^15,16^. BMP-2 functional polypeptides (KIPKASSVPTELSAISTLYL, BMP2pp) were previously synthesized through biosynthesis. BMP2pp has the same receptor binding capacity and osteogenic properties as the BMP-2 protein, and it is more stable and has improved preservation quality compared to the BMP-2 protein^17^.

In this study, we first investigated the effect of titanium (Ti) particles on the osteoblastic differentiation of human placenta-derived mesenchymal stem cells (hPMSCs) in vitro. Then, we examined the effect of the exogenous BMP2pp on Ti-mediated impairments in hPMSCs osteogenesis and the role of macrophage polarization. Finally, we generated a mouse calvarial osteolysis model, verified whether BMP2pp could alleviate Ti-induced bone damage, and further studied the mechanism.

## Results

### Ti particles inhibit the proliferation of hPMSCs

To clarify the effect of Ti particles on hPMSC properties, we compared the expression of specific markers on cells by flow cytometry. As shown in Supplementary Fig. 1a, b, after 3 days of coculture, there was no significant difference between Ti-hPMSCs and healthy hPMSCs. Almost 80% of the cells expressed the mesenchymal markers CD29 and CD90 and did not express the hematopoietic cell surface markers CD34 and CD45. To further delineate the effects of Ti particles on the proliferation of hPMSCs, we first cultured hPMSCs with Ti particles for 3 days to obtain Ti-pretreated hPMSCs (Ti-hPMSCs), removed the Ti particles, and continuously cultured the cells in growth medium for another 4 days. Compared to the healthy hPMSCs at the same time point, Ti-hPMSCs displayed a lower proliferation rate (Supplementary Fig. 2a). This finding demonstrated that although Ti treatment did not affect the stemness of hPMSCs, the proliferation of hPMSCs was impaired.

We also examined the viability of bone marrow-derived macrophages (BMMs) in response to different Ti concentrations. Ti particles inhibited the proliferation of BMMs at concentrations of 0.2 mg/mL and higher (Supplementary Fig. 2b). Therefore, we chose 0.1 mg/mL Ti particles to treat the BMMs and collected the supernatants for further conditioned medium experiments.

### BMP2pp alleviates the impairment of hPMSC osteogenesis induced by Ti particles

To confirm the osteogenic effects of BMP2pp, hPMSCs were induced to differentiate into osteoblasts in the presence of different concentrations of BMP2pp. As the concentration of BMP2pp increased, alkaline phosphatase (ALP) activity and alizarin red S (ARS) staining increased and peaked at a concentration of 100 ng/mL (Supplementary Fig. 3a–c). Similar to the staining results, ALP and Runx2 gene expression was significantly upregulated (Supplementary Fig. 3d, e). The phospho-Smad2/Smad3 and Smad4 protein expression levels were also increased when the cells were treated with BMP2pp (Supplementary Fig. 3f, g), which suggested that BMP2pp promoted osteogenic differentiation in hPMSCs through the BMP/Smad signaling pathway, which was consistent with previous studies^18^.

**Fig. 1:**
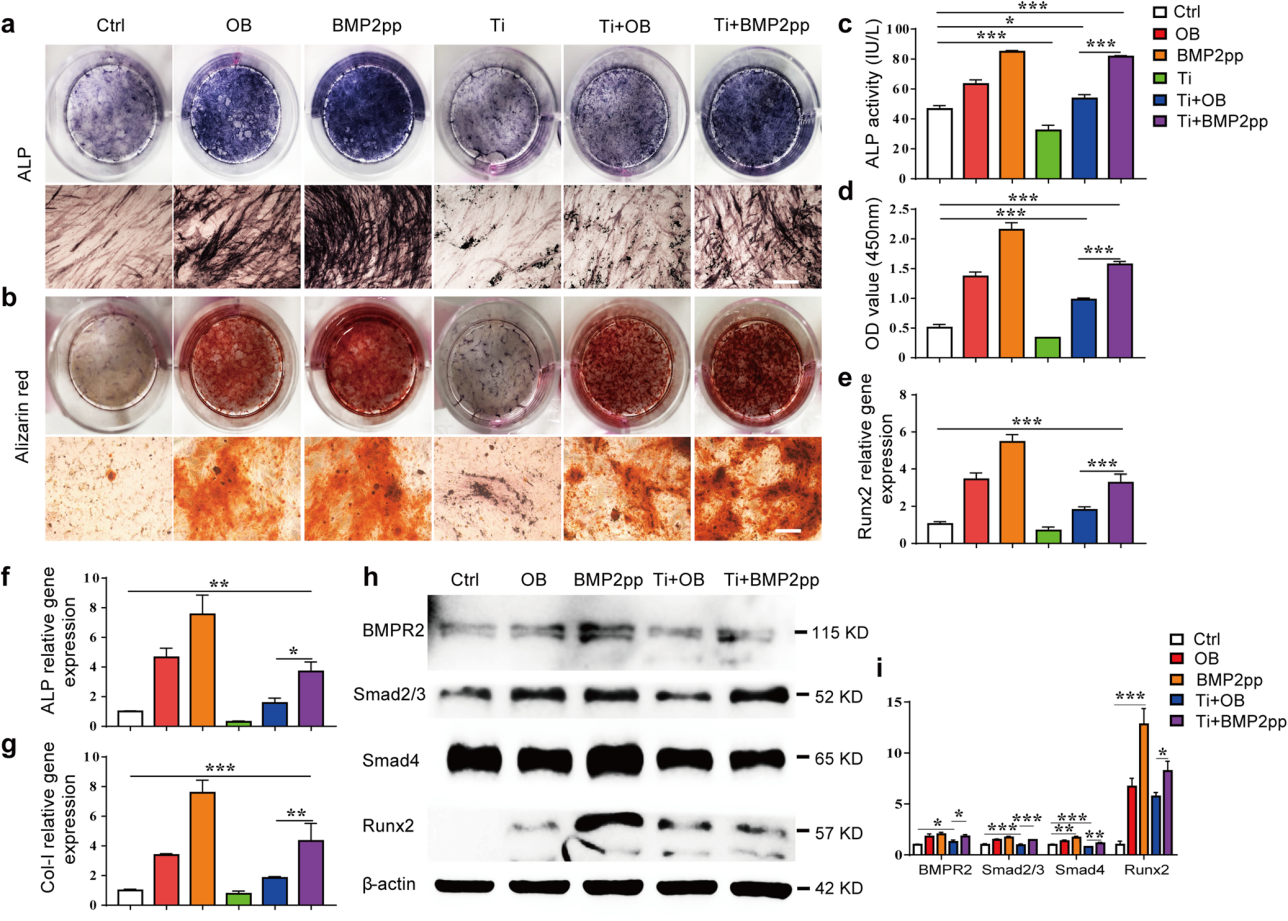
BMP2pp alleviates the impairment of hPMSC osteogenesis induced by Ti particles. **a**, **b** Gross observation (top) and microscope images (bottom) of ALP and alizarin red S (ARS) staining of hPMSC-derived osteoblasts upon different treatment. Scale bars: 200 μm. **c**, **d** Quantitative results of ALP activity and ARS staining. The relative gene expression of **e** Runx2, **f** ALP, and **g** Col-I in hPMSC-derived osteoblasts by qRT‒PCR. **h** The protein expression of BMPR2, Smad2/3, Smad4, and Runx2 in hPMSC-derived osteoblasts by western blot analysis. **i** The relative gray levels corresponding to BMPR2, Smad2/3, Smad4, and Runx2. All the data are presented as the average ± standard deviation (S.D.).

After 7 days of osteogenic induction, ALP and ARS staining showed that Ti particles significantly inhibited the osteoblastic differentiation of hPMSCs. With the addition of BMP2pp, ALP-positive cells, and calcium nodules were significantly increased in the Ti + BMP2pp group (Fig. 1a, b). The ALP activity results were consistent with the ALP staining results (Fig. 1c). The quantification data of ARS staining also revealed that the Ti-BMP2pp group showed more bone mineralization than the OB group (Fig. 1d). The expression of the osteogenic genes ALP, Runx2, and type I collagen (Col-I) was downregulated in Ti-treated cells after 7 days of culture and was recovered in the Ti+BMP2pp group, almost reaching the level of the OB group (Fig. 1e–g). In addition, BMP2pp significantly activated the BMP2 signaling pathway and enhanced the Ti-induced decrease in Runx2 protein expression after 5 days of culture (Fig. 1h, i). Therefore, BMP2pp can significantly ameliorate the inhibition of osteoblastic differentiation and mineralization induced by Ti particles in hPMSCs.

### BMP2pp did not aggravate osteoclastic differentiation in BMMs induced by Ti particles

As reported, a high concentration of BMP-2 could induce osteoclast differentiation in a concentration-dependent manner, which may further promote osteoclast-mediated osteolysis^19–21^. Therefore, we tested the effect of BMP2pp on the osteoclastic differentiation of BMMs. The tartrate-resistant acid phosphatase (TRAP) staining results showed that high concentrations (200 and 400 ng/mL) of BMP2pp could significantly enhance the differentiation of osteoclasts (Supplementary Fig. 4a–c), and upregulate the gene expression of TRAP and osteoclast-associated receptor (OSCAR) (Supplementary Fig. 4d, e). However, a low concentration of BMP2pp (25, 50, and 100 ng/mL) did not significantly promote the differentiation of osteoclasts (Supplementary Fig. 4a–e). The western blot results were consistent with the staining and qRT‒PCR results (Supplementary Fig. 4f). Therefore, we chose 100 ng/mL of BMP2pp for subsequent experiments.

The enhancement of osteoclastic bone resorption caused by Ti particles is one of the reasons for osteolysis around the prosthesis. We first examined the effect of BMP2pp on osteoclast differentiation caused by Ti in vitro. TRAP staining showed that Ti could significantly accelerate the osteoclastogenesis of BMMs. Compared with those in the Ti group, the number and area of osteoclasts were slightly lower in the BMP2pp + Ti group; however, the differences were not statistically significant (Supplementary Fig. 5a–c). The gene expression levels of TRAP and OSCAR were consistent with the staining results (Supplementary Fig. 5d, e). These results suggested that BMP2pp did not aggravate Ti particle-induced osteoclast differentiation in BMMs, which was different from previous data^22,23^.

### BMP2pp impedes the M1 polarization activated by Ti particles

**Fig. 2:**
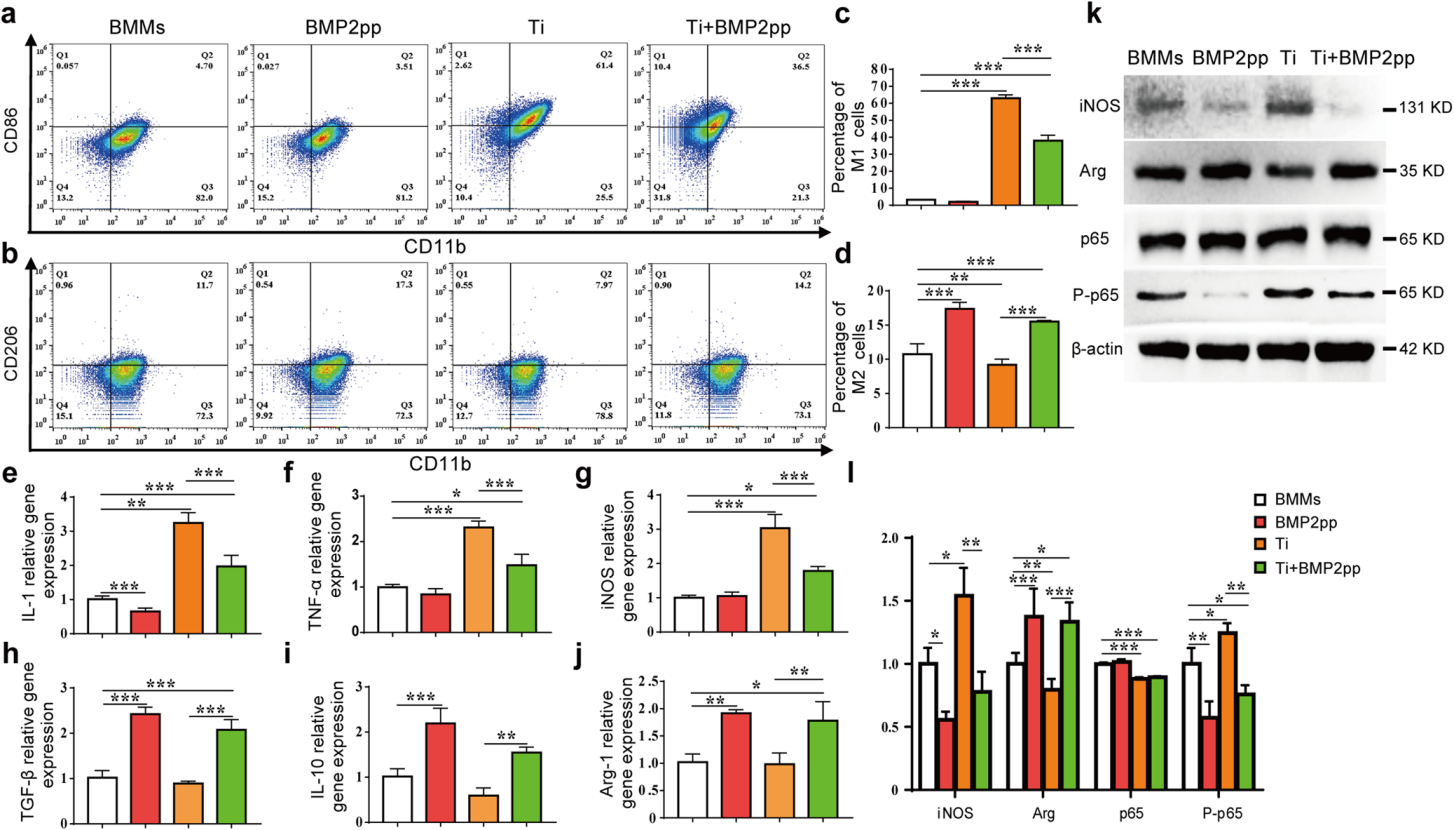
BMP2pp reduced the M1 polarization of macrophages induced by Ti particles. BMMs were treated with Ti particles and/or BMP2pp for 3 days. **a**, **b** The expression of CD11b, CD86, and CD206 of BMMs by FCA. **c**, **d** The percentages of M1 macrophages (CD11b^+^CD86^+^) and M2 macrophages (CD11b^+^CD206^+^) by flow cytometry assay. The relative gene expression of **e** IL-1, **f** TNF-α, **g** iNOS, **h** TGF-β, **i** IL-10, and **j** Arg-1 in BMMs by qRT-PCR. **k** INOS, Arg, p65, and P-p65 protein expression of BMMs by western blot analysis. **l** The relative gray level corresponds to iNOS, Arg, p65, and P-p65. All the data are presented as the average ± standard deviation (S.D.).

Previous studies have shown that macrophages play an essential role in periprosthetic osteolysis^24^. Recently, the immunoregulatory potential of the BMP-2 protein, especially its effect on macrophage polarization, was reported in several studies^25,26^. Therefore, we examined whether BMP2pp regulated macrophage polarization induced by Ti particles. After 3 days of Ti treatment, the percentage of M1 macrophages (CD11b^+^CD86^+^) was significantly higher in the Ti group than in the other groups, and the percentage of M2 macrophages (CD11b^+^CD206^+^) in the BMP2pp group was slightly increased compared with that in the BMMs group. However, the percentage of M1 macrophages was markedly decreased, and the polarization rate of M2 cells was significantly higher in the Ti + BMP2pp group than in the Ti group (Fig. 2a–d). The pro-inflammatory cytokines specific for M1 polarization, including inducible nitric oxide synthase (iNOS), TNF-α, and IL-1, were significantly upregulated in the Ti group (Fig. 2e–g). Moreover, BMP2pp remarkably downregulated the expression of these genes in the Ti+BMP2pp group compared with the Ti group. BMP2pp also increased the expression of anti-inflammatory genes related to M2 polarization, such as TGF-β, Arg-1, and IL-10 (Fig. 2h–j). Consistently, the changes in iNOS and Arginine (Arg) protein levels exhibited a similar trend (Fig. 2k, l). Interestingly, the western blot results demonstrated that BMP2pp treatment did not affect the expression of p65 but inhibited the expression of phospho-NF-κB p65 (p-p65) protein (Fig. 2k, l), which suggested that the activation of the NF-κB signaling pathway in BMMs was inhibited by BMP2pp. In summary, Ti particles shifted macrophages to the M1 phenotype by producing more inflammatory factors. However, BMP2pp can impede the M1 polarization induced by Ti particles and shift macrophages to the M2 phenotype.

### BMP2pp suppresses oxidative stress induced by Ti particles

Reactive oxygen species (ROS) are essential for multiple cellular processes, including cell proliferation, differentiation, apoptosis, and angiogenesis. In addition, ROS production is also critical for M1 polarization^27,28^. Herein, we examined the ROS level of BMMs in the presence of BMP2pp. Compared with those in the control group (Ctrl), ROS levels did not change significantly when BMMs were treated with BMP2pp (BMP2pp group) but increased dramatically in response to Ti stress (Ti group). BMP2pp significantly suppressed the increase in ROS levels induced by Ti (Ti+BMP2pp group) (Supplementary Fig. 6a, b). We further examined the protein levels of superoxide dismutase 2 (SOD2) and malondialdehyde (MDA) in BMMs after the different treatments. As expected, the contents of MDA in BMMs were increased, and SOD2 activity was decreased in the Ti group. Remarkably, BMP2pp reversed the Ti-induced increase in oxidative stress in BMMs in the Ti+BMP2pp group (Supplementary Fig. 6c, d). BMP2pp also significantly upregulated the expression levels of antioxidant genes, including SOD2 and catalase (CAT) (Supplementary Fig. 6e, f). Thus, we examined the expression of the oxidative stress-related proteins NADPH oxidase 2 (NOX2), glutathione peroxidase (GPX4), NIK, and SOD2 in BMMs in the different groups. The changes in the proteins were consistent with the gene expression results (Supplementary Fig. 6g, h). Thus, BMP2pp promotes M2 polarization by reducing oxidative stress induced by Ti particles.

### BMP2pp indirectly enhances the osteogenesis of hPMSCs by regulating macrophage polarization

**Fig. 3:**
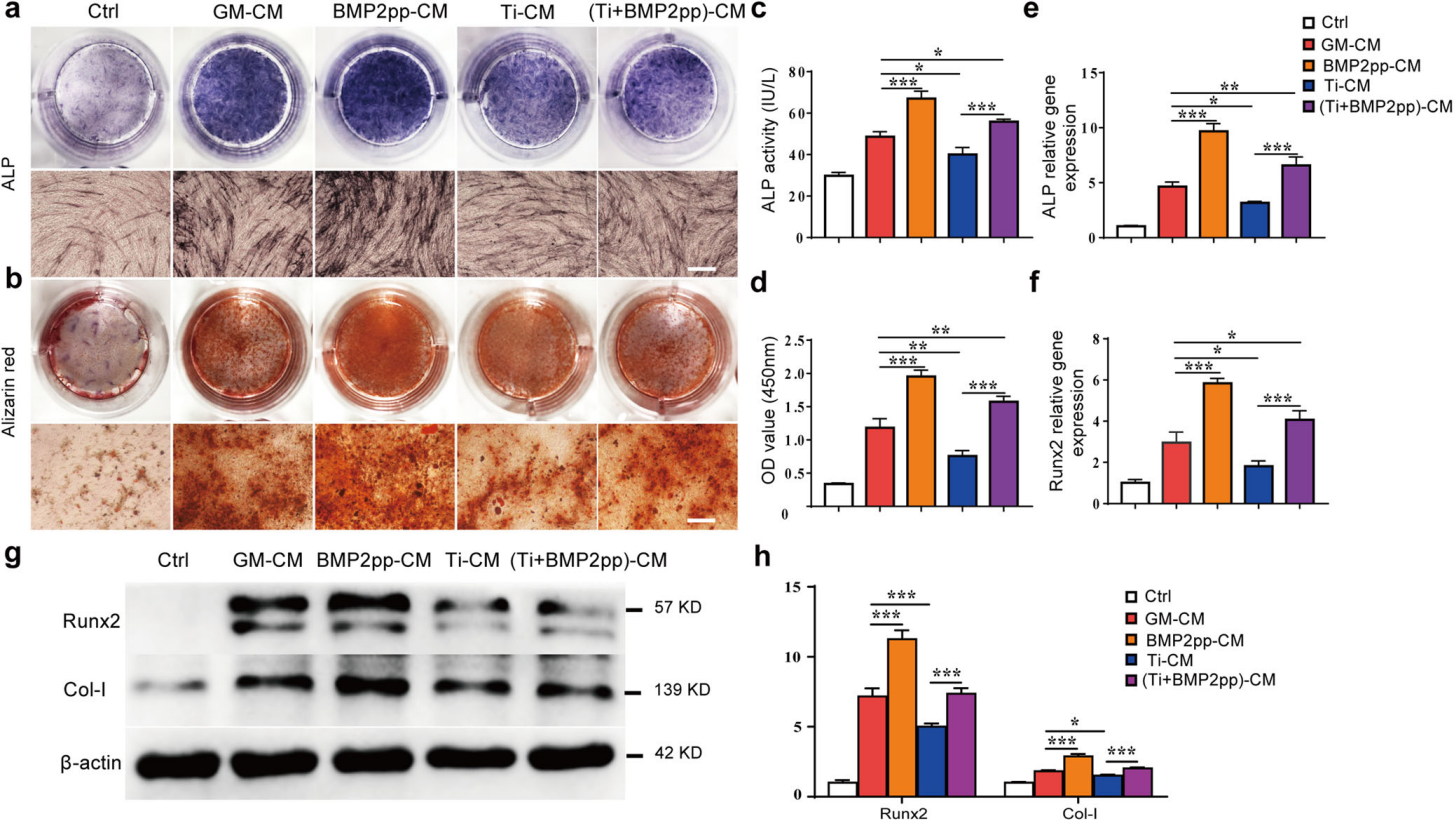
BMP2pp indirectly enhanced the osteogenesis of hPMSCs by regulating macrophage polarization. **a**, **b** General (top) and microscope images (bottom) of ALP (7 days) and ARS (14 days) staining of hPMSC-derived osteoblasts with different conditional mediums. Scale bars: 200 μm. Quantitative analysis of **c** ALP activity, and **d** ARS staining. **e**, **f** ALP/Runx2 gene expression of hPMSC-derived osteoblasts. **g** Expression of osteogenic-related proteins, including Runx2 and Col-I in hPMSCs cultured in the different mediums. **h** The relative gray levels correspond to Runx2 and Col-I. All the data are presented as the average ± standard deviation (S.D.).

BMMs were cultured in a growth medium supplemented with Ti and/or BMP2pp supplement for 2 days, and the supernatant was collected. hPMSCs were cultured in different conditioned media (blending the osteogenic medium (OB medium) with different supernatants at a ratio of 7:3) to induce osteoblasts. ALP and ARS staining showed that, compared with the cells cultured in the conditioned medium (CM) from the Ti-treated macrophage group (Ti-CM), the number of positive cells was significantly increased in the conditioned medium from the Ti+BMP2pp macrophage group ((Ti+BMP2pp)-CM) (Fig. 3a, b). Further quantitative analysis of ALP activity and ARS staining showed consistent changes in staining (Fig. 3c, d). The gene levels of Runx2 and ALP and the protein expression of Runx2 and Col-I were also dramatically increased in cells cultured in conditioned medium from the (Ti+BMP2pp)-CM group compared with those cultured in conditioned medium from the Ti-CM group (Fig. 3e–h). These results demonstrated that BMP2pp could indirectly enhance the osteogenesis of Ti-hPMSCs by regulating macrophage polarization.

### BMP2pp alleviates Ti-induced osteolysis in mice

**Fig. 4:**
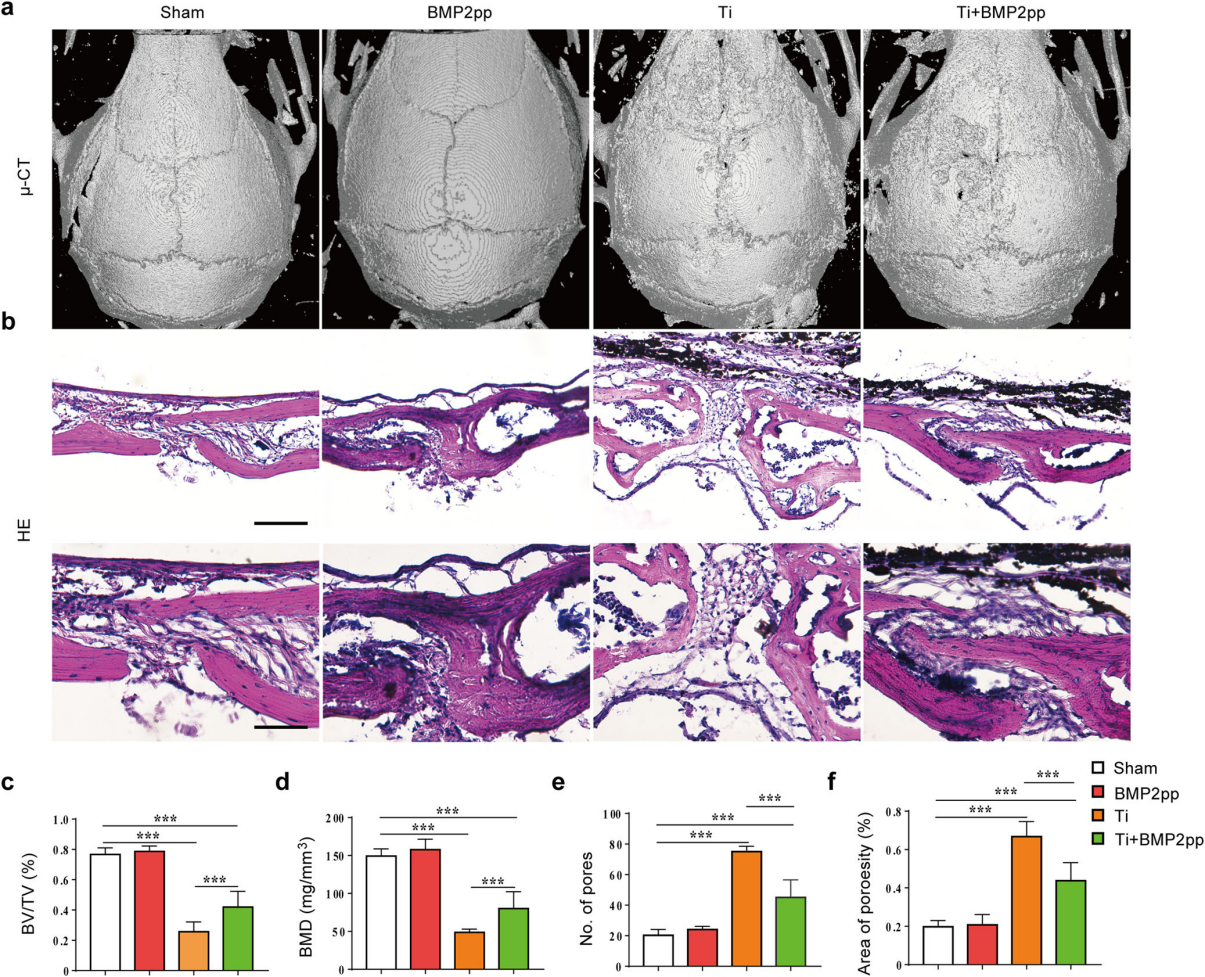
BMP2pp alleviated Ti-induced calvarial bone osteolysis in mice. **a** Micro-CT 3D-reconstructed images of the calvarial bone. **b** Representative images of H&E staining at low (top, scale bars: 100 μm) and high (bottom, scale bars: 50 μm) magnification. Quantitatively evaluate the percentage of **c** BV/TV, **d** BMD, **e** number of osteolytic defects, and **f** the percentage of osteolytic area, *n* = 8. All the data are presented as the average ± standard deviation (S.D.).

We examined whether BMP2pp could alleviate Ti-induced osteolysis in a mouse calvarial osteolysis model. μ-CT scanning and three-dimensional reconstruction results showed that, compared with that in the sham group, apparent bone erosion was observed in the Ti group (Fig. 4a), and the bone volume/tissue volume (BV/TV), bone mineral density (BMD), and trabecular bone thickness (TB.Th) decreased significantly, and the number of pits, pit area, osteoclast surface per bone surface (Oc.S/BS), and eroded bone surface to bone surface (EBS/BS) increased significantly (Fig. 4a, c–f, Supplementary Fig. 7a–c). The phenotype of osteolysis was significantly improved after BMP2pp administration. BMD, BV/TV, and Tb.Th in the Ti+BMP2pp group was significantly increased, and the number of resorption pits, the pit area, Oc.S/BS, and EBS/BS were markedly lower than those in the Ti group. The results of histological H&E staining results were consistent with the μ-CT scanning results (Fig. 4b). BMP2pp had a significant protective effect against Ti-induced osteolysis.

**Fig. 5:**
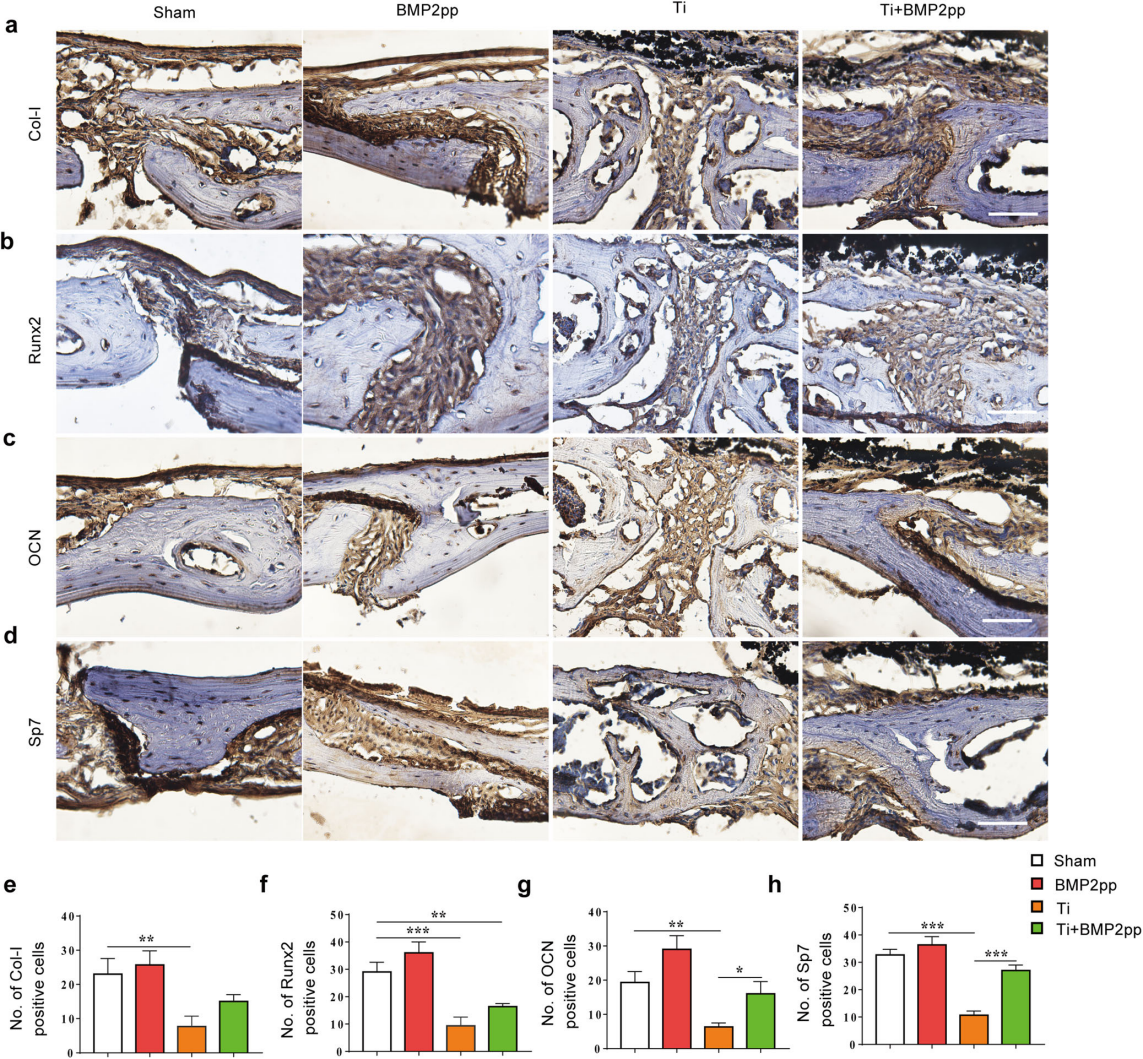
BMP2pp enhanced the calvarial bone formation impaired by Ti particles in mice. Representative images of **a** Col-I, **b** Runx2, **c** OCN, and **d** Sp7 immunohistochemical staining of calvarial bone. scale bars: 50 μm. Number of **e** Col-I, **f** Runx2, **g** OCN, and **h** Sp7 staining positive cells, *n* = 8. All the data are presented as the average ± standard deviation (S.D.).

We then examined the expression of osteogenesis-related proteins, including Col-I, Runx2, osteocalcin (OCN), and Sp7, in calvarial bone tissue by immunohistochemical staining. Compared with that in the sham group, the number of Col-I-, Runx2-, OCN-, and Sp7-positive cells was significantly decreased in the Ti group. However, BMP2pp treatment significantly increased the number of osteoblastic cells in the Ti-BMP2pp group, which was even corrected to a normal extent, similar to that in the sham group (Fig. 5). These results suggested that BMP2pp alleviated Ti-induced osteolysis, which may be partly related to its osteogenic effect.

**Fig. 6:**
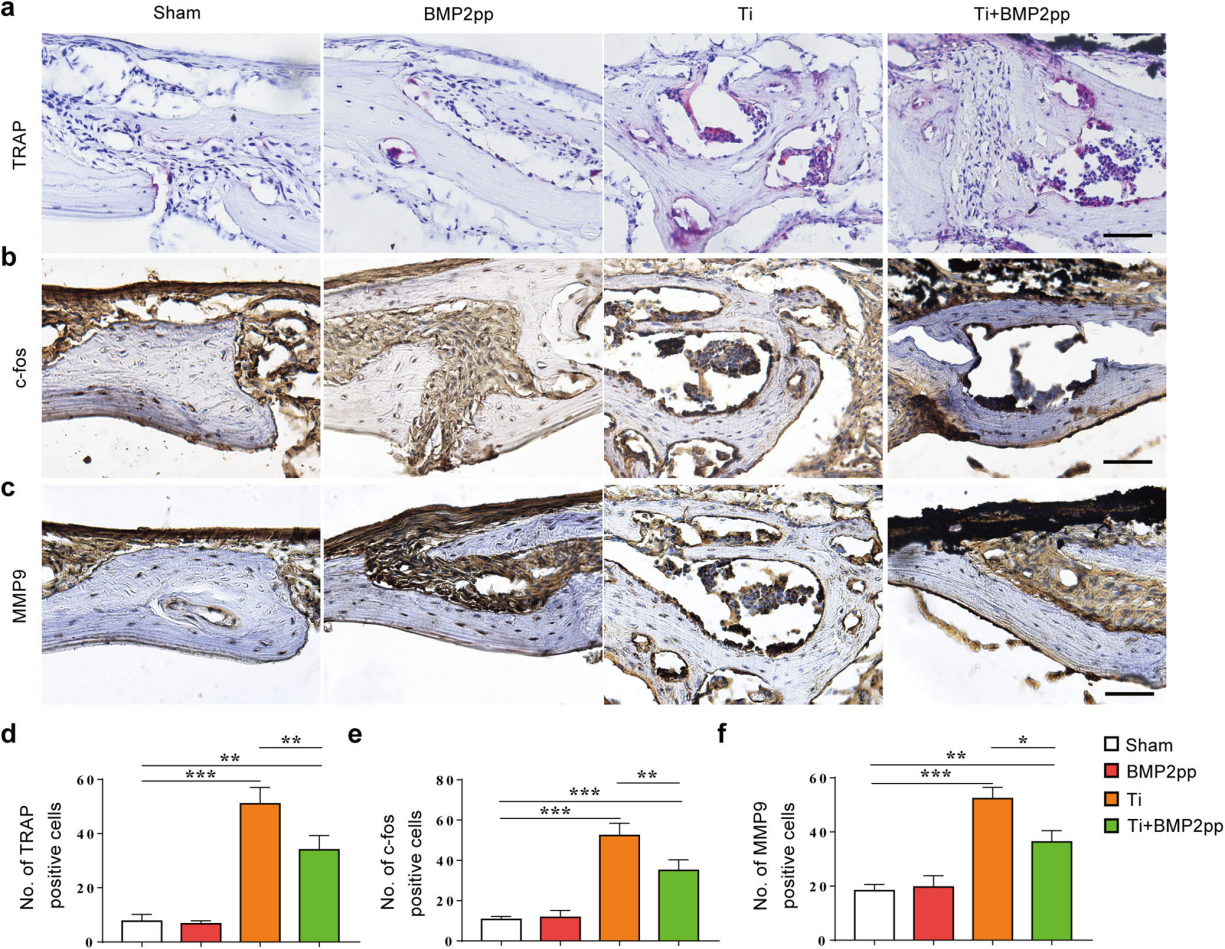
BMP2pp reduced the calvarial bone resorption and inflammatory factor release stressed by Ti particles in mice. **a** Representative images of TRAP histological staining. Images of **b** c-fos, and **c** MMP9 immunohistochemical staining of calvarial bone. scale bars: 50 μm. The number of **d** TRAP, **e** c-fos, and **f** MMP9 staining positive cells, *n* = 8. All the data are presented as the average ± standard deviation (S.D.).

Then, we analyzed the effects of BMP2pp on the calvarial bone resorption and inflammatory factor release by TRAP and immunohistochemical staining. As shown in Fig. 6, compared with that in the sham group, the number of TRAP-positive osteoclasts and c-fos-, and metallopeptidase 9 (MMP9)-positive cells in the calvarial bone of the Ti group was significantly increased. The addition of BMP2pp significantly reduced the number of osteoclasts and c-fos-, and MMP9-positive cells in bone tissue.

**Fig. 7:**
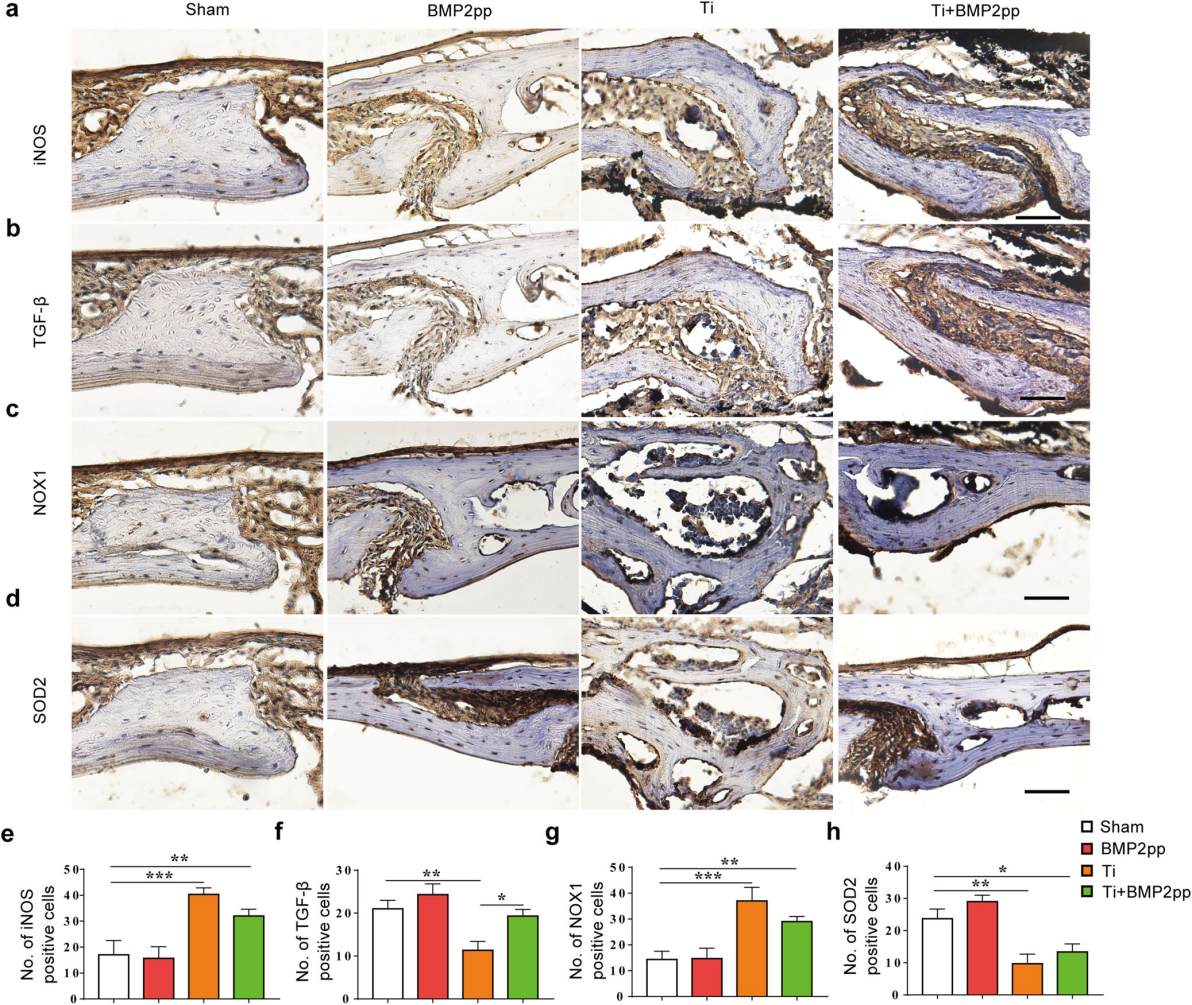
BMP2pp promoted M2 polarization of macrophages and enhanced antioxidant capacity in the calvarial bone caused by Ti particles in mice. Representative images of immunohistochemical staining of **a** iNOS, **b** TGF-β, **c** NOX1, and **d** SOD2. scale bars: 50 μm. The number of **e** iNOS, **f** TGF-β, **g** NOX1, and **h** SOD2 positive cells, *n* = 8. All the data are presented as the average ± standard deviation (S.D.).

To further investigate the effects of BMP2pp on macrophage polarization in vivo, we examined the expression of iNOS and TGF-β in the calvarial bone (Fig. 7a, b). The number of iNOS-positive cells was significantly increased in the Ti group. In contrast, TGF-β-positive cells decreased, suggesting that macrophages were more polarized to the M1 type, perhaps contributing to Ti-induced calvarial osteolysis. However, BMP2pp alleviated the change in M1 polarization caused by Ti particles, and TGF-β-positive expression cells were significantly increased (Fig. 7e, f).

Finally, we examined the expression of NOX1 and SOD2 in bone tissue. The number of NOX1-positive cells was markedly increased in the Ti group, while the number of SOD2-positive cells decreased significantly, suggesting that the level of oxidative stress increased significantly with Ti stress in mice with calvarial osteolysis. However, the administration of BMP2pp reduced the enhancement of oxidative stress levels caused by Ti (Fig. 7c, d, g, h).

These results showed that BMP2pp could alleviate Ti-induced osteolysis in vivo. In addition to promoting osteoblast differentiation, BMP2pp can also ameliorate the high oxidative stress microenvironment and further inhibit the polarization of M1 macrophages caused by Ti particles. BMP2pp may be useful for preventing wear debris-induced osteolysis.

## Discussion

rhBMP-2 is widely used in the clinic for its excellent osteogenic effects. However, rhBMP-2 still has the defect of explosive release in the biological environment in the early stage. Almost 10^3^-fold higher than physiological levels, the release of this high concentration of rhBMP2 may cause many adverse side effects, such as pain, edema, poor bone structure, and excess inflammation^16,29,30^. In addition, because the concentration of rhBMP-2 soon drops below the treatment level, it is difficult to continuously promote osteogenesis. To solve this problem, an increasing number of researchers have focused on developing effective rhBMP-2 release-controlled delivery strategies to use in clinical bone tissue engineering. However, natural proteins may fold when they adhere to the material’s surface, and insufficient exposure to active sites will lead to low biological activity^30–32^. Polypeptide is a continuous chain of 20–50 amino acids. Synthetic functional polypeptides have shown great promise for biomedical applications and might be good candidates instead of recombinant proteins.

Typically, rhBMP-2 contains 115 amino acids, and BMP2pp is the functional peptide of BMP-2, which contains 20 amino acids. Compared with proteins, polypeptides are low-cost and can be synthesized by various chemical methods. The functional active sites of polypeptides can be fully exposed and bind with cell surface receptors. Moreover, the chemical diversity of side chains and the ability of synthesized polypeptides to form secondary structures enable their broad applications in the field of surface modification in tissue engineering^33,34^. In addition, compared with high molecular weight proteins, the biological function of the polypeptide is more stable and durable. Therefore, polypeptides are easily synthesized and exhibit improved stability in composites with materials. The ALP and ARS staining and qRT‒PCR results confirmed that 100 ng/mL of BMP2pp significantly induced osteogenesis in hPMSCs. BMP-2 also directly affects osteoclast differentiation, fusion, and survival by working synergistically with the RANK/RANKL signaling pathway^35,36^. The TRAP staining results showed that high concentrations (200 and 400 ng/mL) of BMP2pp could significantly enhance the differentiation of osteoclasts. However, we did not find the same promotion of osteoclastogenesis in response to low concentrations of BMP2pp (25, 50, and 100 ng/mL). Several studies have shown that BMP-2 overexpression in RAW264.7 cells or exogenous addition of rhBMP-2 could increase Ti particle-induced osteoclast differentiation in RAW264.7 cells in the presence of RANKL^22,23^. However, we did not find that BMP2pp could aggravate the osteoclast differentiation of BMMs induced by Ti particles. This may be due to the concentration of BMP2pp, the different sources of macrophages we used, or even the different effects of rhBMP-2 and BMP2pp. The effects and potential mechanisms of BMP2pp on osteoclasts still need to be further explored.

Recently, many studies have identified the inhibitory effects of wear particles on osteogenesis^37^. Wear particles can directly damage the activity of ALP and the expression of various collagens in osteoblasts, resulting in a decrease in osteoblast differentiation and mineralization^38^, which is consistent with our experimental results. The osteoblastic differentiation of hPMSC was inhibited when they were cultured with Ti particles. In addition, the basic activities of the Wnt and BMP signaling pathways were inhibited in osteoblast precursor cells treated with Ti particles^39,40^. BMP-2 can bind to the type II receptor (BMPRII) on target cells, recruit phosphorylated Smad2/3, form a polymer with Smad4, enter the nucleus to mediate transcription, and promote osteoblast differentiation. BMP-2 can also regulate the ligands of the Wnt signal transduction pathway to promote β-catenin expression in cells, further promoting osteoblast differentiation^41^. Therefore, rhBMP-2 may be a candidate for treating peri-implant osteolysis. In vitro, the BMP/Smad signaling pathway osteogenesis was activated in hPMSCs treated with BMP2pp, and the inhibition of osteoblastic differentiation and mineralization in hPMSCs induced by Ti particles was rescued. In vivo, compared with that in the mouse calvarial bone osteolysis group, BMP2pp treatment significantly increased the BMD and BV/TV and reduced the porosity of mouse calvarial bone, which indicated that BMP2pp might be a potential therapy for osteolysis.

Macrophages play an important role in the immune system, and they are involved in a variety of diseases. The chronic foreign body response to wear particles is also mediated by macrophages. Macrophages recognize and phagocytize wear particles, are activated, and then secrete various inflammatory cytokines (e.g., TNF-α, IL-1β, and IL-6) and chemokines (e.g., C-C motif chemokine ligand 2 (CCL2), C-X-C motif chemokine ligand 9 (CXCL9) and CXCL10). These factors recruit more macrophages to the injury site to disturb tissue homeostasis and stimulate other stromal cells^42^. The local inflammatory microenvironment further induces macrophage polarization toward the M1 phenotype through different pathways, including the JNK, p38 ɑ/β MAPK, and NF-kB signaling pathways^43–45^. This sustained proinflammatory state also drives osteoclast activation at the bone-implant interface, which increases bone destruction and impairs the stability and lifespan of the implant^46^. Wear particles and the inflammatory microenvironment induce M1 macrophage polarization, aggravating the production of proinflammatory mediators. This further promotes the formation of osteoclasts^47^. Our experiments also confirmed that Ti particles could promote the polarization of macrophages to the M1 type. In contrast, M2 macrophages have anti-inflammatory effects and can produce anti-inflammatory factors to reduce these adverse effects^48^. Therefore, limiting the effects of M1 polarizing factors in the interface tissue and promoting the M2 polarization of macrophages may be a strategy to protect against osteolysis of wear particles and their immunomodulate effects and as a promising means to prolong the life of implants^49^.

To simultaneously enhance the differentiation of MSCs to osteoblasts and induce the polarization of macrophages to the M2-type, there have been several studies on the co-administration of BMP-2 and immunomodulatory cytokines (IL-4) or metal ions (Zn^2+^) on Ti implants. These dual-modified implants could create a favorable osteoimmune microenvironment by switching macrophages from the M1 to M2 phenotypes at the bone-implant interface and improve bone regeneration and osseointegration for successful implantation^50,51^. Interestingly, the results also showed the synergistic effect of IL-4 or Zn^2+^ combined with BMP-2 (rhBMP-2 or BMP-2 peptide) on M2-type macrophage polarization. In our previous work, we grafted the cell anchoring RGD motif and BMP-2 peptide onto Ti substrates. We found that dual-functionalized Ti substrates promoted the adhesion and osteogenesis of MSCs and conspicuous immunoregulation to shift macrophages to alternative (M2) phenotypes and inhibit inflammation^52^. These data suggest that BMP-2 is associated with M2 macrophages, but the specific mechanism remains unclear. There have been few studies on the direct effect of BMP-2 on macrophages, and our study showed that BMP2pp could significantly reduce the polarization of M1 macrophages and increase M2 macrophage polarization. The polarization of macrophages is related to multiple signaling pathways, such as NF-κB, JAK2/STAT3, ROS/ERK, and mTOR. The NF-κB pathway participates in innate and adaptive immunity, and its activation is an essential factor in inducing macrophages to differentiate into the M1 phenotype^53^. In vitro, the protein expression of NIK and phosphorylated p65 in BMMs was significantly increased when BMMs were cultured with Ti particles, which indicated that the NF-κB pathway was activated in BMMs. In addition, we also found that Ti particles promoted oxidative stress in BMMs. Redox homeostasis involves the balance between ROS and antioxidant systems. Increased oxidative stress can activate the NF-κB p65 pathway, and ROS are upstream signals of p65 activation^54^. The ROS levels in BMMs were enhanced when the cells were stimulated with Ti particles. BMP2pp treatment significantly enhanced the expression of antioxidant genes, including SOD2 and CAT, increased the activity of the antioxidant enzyme SOD2, and reduced the expression of oxidative stress-related proteins, such as NIK and NOX2. Thus, BMP2pp inhibited the oxidative stress and the NF-κB p65 pathway in BMMs activated by Ti particles and promoted M2 macrophage polarization.

To further confirm the relationship between macrophage polarization and osteolysis, we used the supernatant of macrophages cultured under different culture conditions as a conditioned medium to observe their effect on the osteogenic differentiation of hPMSCs^55,56^. We found that the conditioned medium from BMP2pp-treated macrophages significantly promoted the osteogenic differentiation of hPMSCs, which suggested that BMP2pp could indirectly alleviate the Ti-induced decrease in osteogenic differentiation in hPMSCs by promoting the polarization of macrophages to the M2 type and the secretion of related factors.

Increasing the service life of joint prostheses remains a challenging problem. There are still many limitations in our study. The release of particles is continuous for a long time in the clinic. However, we only treated calvarial osteolysis with a single injection of free BMP2pp and collected samples two weeks later to observe the curative effect. In addition, the composition and microstructure of calvarial bone and long bone are different^57^. In the future, we will try to compound BMP2pp with implant materials and use a large animal osteolysis model to further confirm the inhibitory effect of BMP2pp on wear particle-induced osteolysis.

**Fig. 8:**
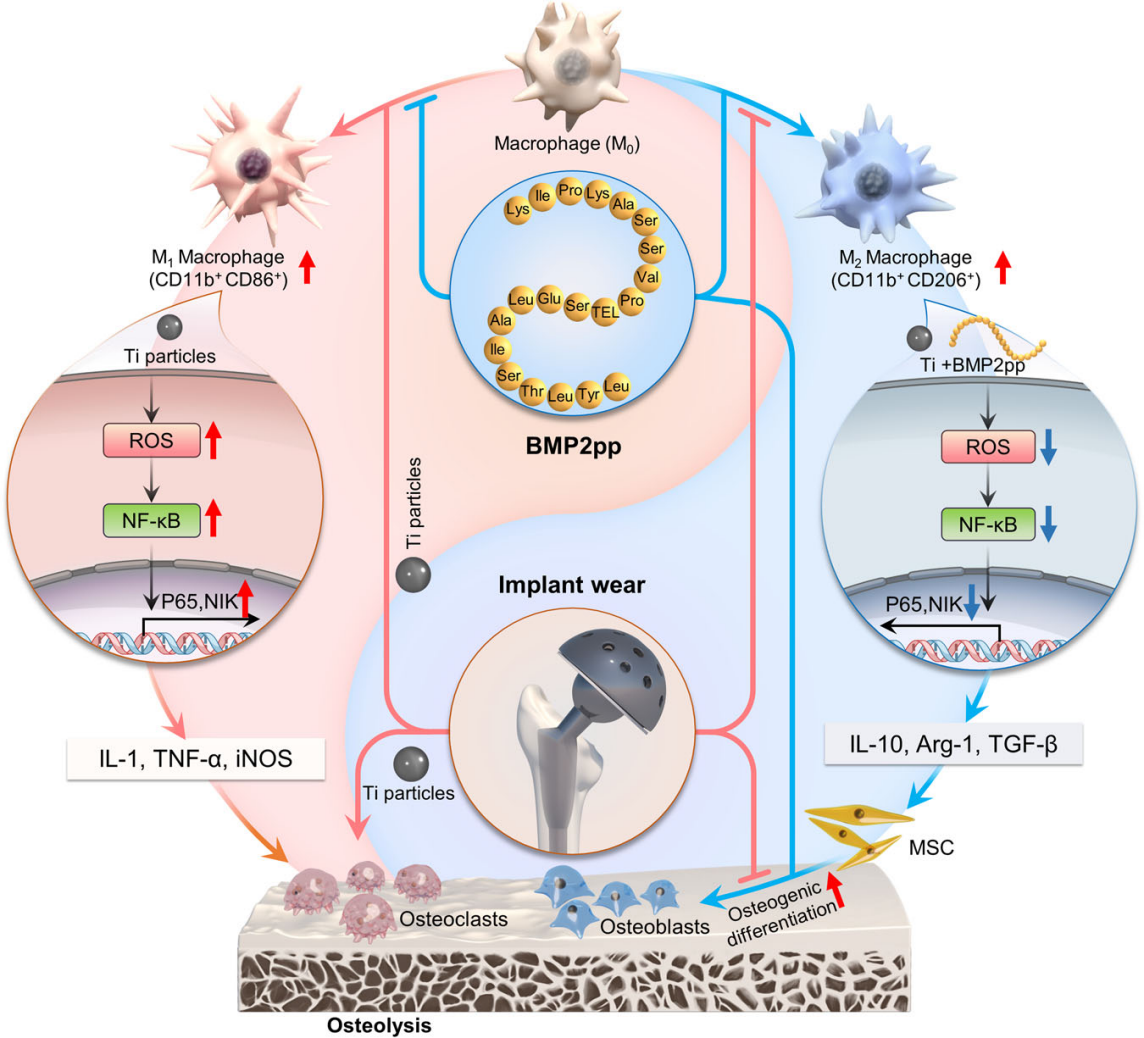
Schematic illustrations of the dual effects of BMP2pp in Ti particles induced osteolysis. In addition to directly promoting the osteogenic differentiation, BMP2pp also can inhibit the polarization of M1 macrophage caused by Ti particles, and promote the polarization of macrophages to M2 type, which secret related factors to reduce the enhanced oxidative stress in the microenvironment by Ti. Thus, BMP2pp could alleviate Ti-induced osteolysis. This image does not contain any third-party material or previously-created elements.

In summary, the inflammatory factors stimulated by Ti particles inhibit the differentiation of osteoblasts. BMP2pp directly promotes osteogenic differentiation and alleviates Ti-induced osteogenic inhibition. Moreover, BMP2pp can promote the polarization of macrophages to the M2 type, which secretes related factors to reduce the Ti-induced oxidative stress in the microenvironment and indirectly alleviates Ti-induced osteolysis (Fig. 8). Overall, our results indicate that BMP2pp may be a therapeutic candidate for the prevention and treatment of wear debris-induced osteolysis in the clinic.

## Methods

### Ethics statement for human and animal objects

Written informed consent was obtained from healthy donors, and discarded human placental tissues were acquired according to the approved protocol of the Institutional Review Board (IRB) at the First Affiliated Hospital of Soochow University. All animal experimental protocols were approved by the Animal Care and Use Committee of Soochow University. All animal experiments were performed in accordance with the associated relevant guidelines and regulations for working with live vertebrate animals.

### Materials

Commercial titanium (Ti) particles were obtained from Alfa Aesar (#000681, Heysham, UK) and prepared as previously reported^58^. In short, we first sterilized the Ti particles at 180 °C for 6 hours and then immersed them in 75% ethanol to remove residual endotoxins. The particles were dried and irradiated by UV for further use. Based on our previously described peptide synthesis strategy^59^, BMP2pp (KIPKASSVPTELSAISTLYL) was synthesized with assistance from ChinaPeptides Co., Ltd., (Shanghai, China). The peptides were dissolved and stocked in dimethyl sulfoxide (DMSO) at a concentration of 1 mg/10 μL.

### Cell isolation, culture, and conditioned medium preparation

hPMSCs were isolated and cultured as described previously^60^. In brief, cells were isolated from the central placental cotyledons and cultured in low glucose Dulbecco’s modified Eagle’s medium (DMEM) (Sigma‒Aldrich, USA) supplemented with 10% heat-inactivated fetal bovine serum (FBS, Gibco, USA), 1% antibiotics (100 U/mL penicillin, 100 μg/mL streptomycin) and 2 mmol/L glutamine (Sigma‒Aldrich, USA). The cultures were maintained at 37 °C with 5% CO_2_, and the medium was replaced 1–2 times each week. After three passages, phenotypic analysis was performed by flow cytometry.

A total of 5 × 10^4^ cells were seeded into each well of 12-well plates and cultured in growth medium (GM, a-MEM supplemented with 10% FBS and 1% antibiotic) with Ti particles (0.1 mg/mL) for 3 days. Then, the Ti particles were removed to obtain Ti-pretreated hPMSCs (Ti-hPMSCs), and the effects of Ti particles on hPMSCs were examined. GM supplemented with 50 μg/mL ascorbic acid and 10 mM β-glycerol phosphate was used as osteogenic medium (OB medium). To explore the effects of Ti particles on osteoblastic differentiation, hPMSCs, and Ti-hPMSCs were cultured in OB medium with or without 100 ng/mL BMP2pp, and hPMSCs cultured in GM was used as the control group (Ctrl).

Femurs and tibiae bone marrow cells were extracted from 6-week-old C57/BL6 mice as described previously^61^. Briefly, the cells were cultured overnight, and the nonadherent cells were harvested. Bone-marrow-derived macrophages (BMMs) were obtained after the cells were cultured in macrophage induction medium (GM supplemented with 10 ng/mL macrophage-colony stimulating factor (M-CSF; Peprotech, USA) for 72 hours.

BMMs were cultured in six-well plates (2 × 10^5^/well). After 6 hours, Ti particles (0.1 mg/mL) were added to the GM with or without BMP2pp (100 ng/mL). After 2 days of culture, the supernatants were collected and mixed with OB medium at a ratio of 3:7 to obtain conditioned medium (CM), which included GM-CM, BMP2pp-CM, Ti-CM, and (Ti+BMP2pp)-CM. hPMSCs were cultured in the different CMs to induce osteoblasts in the GM-CM, BMP2pp-CM, Ti-CM, and (Ti+BMP2pp)-CM groups. Moreover, cells cultured in the GM were used as the control group (Ctrl).

### Cell viability

BMMs (1 × 10^4^/well, 96-well plates) were cultured with gradient concentrations of Ti particles (0.025, 0.05, 0.1, 0.2 mg/mL) for 24, 72, and 120 hours. Ten microliters of cell counting kit-8 reagent (CCK-8, Tongren Chemistry, Japan) was added to each well at each time point and incubated at 37 °C for another 2 hours. The optical density (OD) was measured by a microplate reader (BioTek, USA) at an absorbance of 450 nm.

### Osteoblast differentiation and mineralization staining

As described previously, to confirm the effects of BMP2pp on the osteoblast differentiation and mineralization of hPMSCs, the cells were stained for alkaline phosphatase (ALP) 7 days after osteogenic induction, or alizarin red S (ARS) staining was performed after the cells were cultured in OB medium for 14 days^61^. The staining images were taken under a microscope (Carl Zeiss, Germany). An ALP assay kit (Beyotime Biotech, China) was used to determine the activity of ALP. The absorbance at 562 nm was measured by a microplate reader. Then, 5% perchloric acid was used to extract the Alizarin red dye, and the absorbance at 490 nm was measured by a microplate reader.

### Osteoclastic differentiation

BMMs (1 × 10^4^ cells/well, 96-well plate) were cultured in osteoclast induction medium (GM supplemented with 30 ng/mL M-CSF and 50 ng/mL RANKL, R&D Systems) with different concentrations of BMP2pp (25, 50, 100, 200, 400 ng/mL) for 5 days. The cells were then stained for TRAP (Sigma‒Aldrich, USA). Images of TRAP-positive multinucleated (nuclei ≥ 3) cells were obtained under a microscope.

### Flow cytometry assay (FCA)

hPMSCs were treated with or without Ti particles (0.1 mg/mL) for 5 days. The cells were collected and stained with anti-human antibodies (eBioscience, FITC-CD29 (11-0299-42), PerCP/Cy5.5-CD90 (45-0909-42), PE-CD34 (12-0349-42), PE-CD45 (12-0459-42), dilution ratio 1:1000) or isotype control antibody. Cell surface markers were measured by FCA (Merck Millipore, Germany).

To study the effects of BMP2pp on macrophage polarization, BMMs (1 × 10^5^/well, six-well plates) were treated with 0.1 mg/mL Ti particles and cultured with or without 100 ng/mL BMP2pp. The cells were harvested after 3 days of culture and then incubated with anti-mouse antibodies (eBioscience, APC-CD11b (17-0112-82), PE-CD86 (12-0862-82), PE-CD206 (12-2061-82), dilution ratio 1:1000) or isotype control antibody. The cells were analyzed by FCA. CD11b^+^CD86^+^ cells were defined as M1 macrophages, while CD11b^+^CD206^+^ cells were defined as M2 macrophages.

### Oxidative stress assay

BMMs were first treated as described for macrophage polarization for 24 hours. To determine the level of reactive oxygen species (ROS), cells were treated with 10 μM 2’,7’-dichlorofluorescein diacetate (DCFH-DA, Beyotime, China) and incubated at 37 °C for 30 minutes. After being washed with PBS, the cells were observed using a microscope. The activities of total superoxide dismutase (SOD) and malondialdehyde (MDA) were analyzed by SOD and MDA assay kits (Jiancheng, China).

### Quantitative real-time PCR (qRT‒PCR)

Total RNA was extracted from the cells according to the instructions of the TRIzol kit (Beyotime, China), reverse transcribed, and then subjected to qRT‒PCR analysis (Bio-Rad, USA). The relative gene expression was calculated by the 2^−ΔΔCt^ method. GAPDH was used to normalize changes in target gene expression. The primer sequences of the human and mouse genes are listed in Supplementary Tables 1, 2.

### Western blot analysis

Total protein was extracted from cells with RIPA lysis buffer (NCM Biotech, China), and the protein concentration was determined with a BSA protein detection kit (Beyotime, China). Proteins were separated on a 12% SDS‒PAGE gel and transferred onto polyvinylidene fluoride membranes. The membranes were incubated with primary antibodies (Abcam, USA) overnight at 4 °C, including Runt-related transcription factor 2 (Runx2, ab236639, dilution ratio 1:1000), NF-κB-inducing kinase (NIK, ab203568, dilution ratio 1:500), p65 (ab32536, dilution ratio 1:1000), P-p65 (ab76302, dilution ratio 1:1000), BMP receptor type 2 (BMPR2, ab130206, dilution ratio 1:1000), Smad2/3 (ab202445, dilution ratio 1:1000), P-smad2/3 (ab254407, dilution ratio 1:1000), Smad4 (ab230815, dilution ratio 1:1000), iNOS (ab283655, dilution ratio 1:1000), arginine (Arg, ab203490, dilution ratio 1:1000), type I collagen (Col-I, ab138492, dilution ratio 1:5000), c-fos (ab222699, dilution ratio 1:1000), TRAP (ab52750, dilution ratio 1:5000), p38 (ab170099, dilution ratio 1:1000), P-p38 (ab4822, dilution ratio 1:1000), NADPH oxidase 2 (NOX2, ab129068, dilution ratio 1:1000), glutathione peroxidase (GPX4, ab125066, dilution ratio 1:1000), SOD2 (ab68155, dilution ratio 1:1000) and β-actin (ab8226, dilution ratio 1:1000). After being washed, the membranes were incubated with the corresponding secondary antibodies (goat anti-mouse, ab205719, goat anti-rabbit, ab205718, dilution ratio 1:10,000) for 2 hours. The target proteins were visualized using ECL reagents (Thermo Fisher Scientific, USA), and digital images were taken using a ChemiDoc™ Touch Imaging System (Bio-Rad Laboratories, USA). All blots were processed in parallel and derive from the same experiment. Band intensity was quantified using ImageJ (National Institutes of Health, USA).

### Ti particle-induced calvarial osteolysis in mice

As previously described, a murine calvarial osteolysis model was generated^62^. In short, 7-week-old male C57BL/6 mice were first randomly divided into four groups (*n* = 8/group), including two control groups (sham and BMP2pp) and two Ti particle-induced calvarial osteolysis groups (Ti and Ti+BMP2pp). The mice were anesthetized with 50 mg/kg pentobarbital via intraperitoneal injection, the skin of the cranium was incised, and the cranial periosteum was removed. Forty microliters of PBS with or without 0.1 mg/mL BMP2pp (sham and BMP2pp groups, respectively), and 40 μL of PBS containing Ti particles (500 mg/mL) with or without 0.1 mg/mL BMP2pp (Ti and Ti+BMP2pp groups, respectively) were embedded in the middle of the calvarial bone. Mice received a subcutaneous injection of 0.5 mg/kg buprenorphine to control pain. The mice were given food and water normally after the operation. All mice were euthanized by CO_2_ inhalation and secondary cervical dislocation two weeks after the surgery, and the calvarial bones were dissected free of soft tissue.

### Microcomputed tomography (μ-CT) scanning

Calvarial bone (*n* = 5/group) was analyzed by μ-CT scanning (SkyScan 1176, SkyScan, Belgium, 65 kV, 385 mA, and 18 mm resolution). To avoid metal artifacts, wear particles were removed before scanning. Images were reconstructed using the software provided by the manufacturer. In short, raw data obtained from μ-CT scanning were reconstructed with NRecon software and then reoriented with Dataviewer software (Bruker, Aartselaar, Belgium) to generate two-dimensional coronal images of μ-CT slices. The circular region of interest (ROI) with a diameter of 3 mm was selected, and CTAn software (SkyScan, Aartselaar, Belgium) was utilized to generate three-dimensional (3D) images and calculate the bone volume/tissue volume (BV/TV), bone mineral density (BMD), the ratio of the eroded bone surface to the bone surface (EBS/BS), the average trabecular bone thickness (TB.Th, mm) and osteoclast surface per bone surface (Oc.S/BS). 3D μ-CT images were visualized using Mimics v10.01 software (Materialise, Leuven, Belgium). The number of pores and area of porosity within the ROI were counted by ImageJ software as previously described^63^.

### Histological and immunohistochemical staining

The calvaria bones were fixed, decalcified, embedded in paraffin, and then sliced. Hematoxylin-eosin (H&E) staining was performed to examine general histopathological changes. TRAP staining was used to identify osteoclasts. Images were taken centered on the midline suture of the sections under a microscope. Positive TRAP localization was quantified by counting the pixel area in the ROI in five consecutive slices by using ImageJ.

After regular deparaffinization, 0.3% hydrogen peroxidase incubation, and antigen retrieval, the slices were subjected to immunohistochemical staining. The sections were blocked with normal goat serum and then incubated with primary anti-mouse antibodies (Abcam, USA, Col-I (ab138492, dilution ratio 1:1000), Runx2 (ab192256, dilution ratio 1:1000), osteocalcin (OCN, ab93876, dilution ratio 1:1000), Sp7 (ab209484, dilution ratio 1:1000), c-fos (ab283655, dilution ratio 1:2000), MMP9 (ab76003, dilution ratio 1:1000), iNOS (ab283655, dilution ratio 1:2000), TGF-β (ab215715, dilution ratio 1:500), NOX1 (ab131088, dilution ratio 1:1000) and SOD2 (ab68155, dilution ratio 1:250)) and biotinylated secondary antibodies (goat anti-rabbit, ab205718, 1:2000). Finally, a brown color appeared when the sections were incubated with avidin-biotin enzyme reagent. Images were taken with a microscope, and the results were analyzed by ImageJ. The number of positively stained cells was evaluated in five randomly selected visual fields.

### Statistical analysis

The data are presented as the average ± standard deviation (S.D.) with no less than three replicates for each experimental condition. Student’s *t* test was used to assess the significant differences between two groups, and one-way ANOVA followed by Tukey’s post hoc test was used for multiple comparisons. GraphPad Prism software (GraphPad Software, Inc., La Jolla, CA) was used for statistical analyses. *P* values < 0.05 were considered significant. Statistical differences are illustrated in the figures as *p < 0.05, **p < 0.01, ***p < 0.001.

### Reporting summary

Further information on research design is available in the Nature Research Reporting Summary linked to this article.

## Supplementary information

Supplementary Material

Reporting Summary

## Data Availability

The data used to support the findings of this study are available from the corresponding author on request.
