# Supplementary material for: BMP-2 functional polypeptides relieve osteolysis via bi-regulating bone formation and resorption coupled with macrophage polarization
Source: NPJ Regen Med. 2023 Feb 9;8:6. doi: 10.1038/s41536-023-00279-2 (PMC9911742; doi:10.1038/s41536-023-00279-2)
Supplement: Supplementary file 1 — Supplementary Material [file 41536_2023_279_MOESM1_ESM.pdf]

Supplementary material

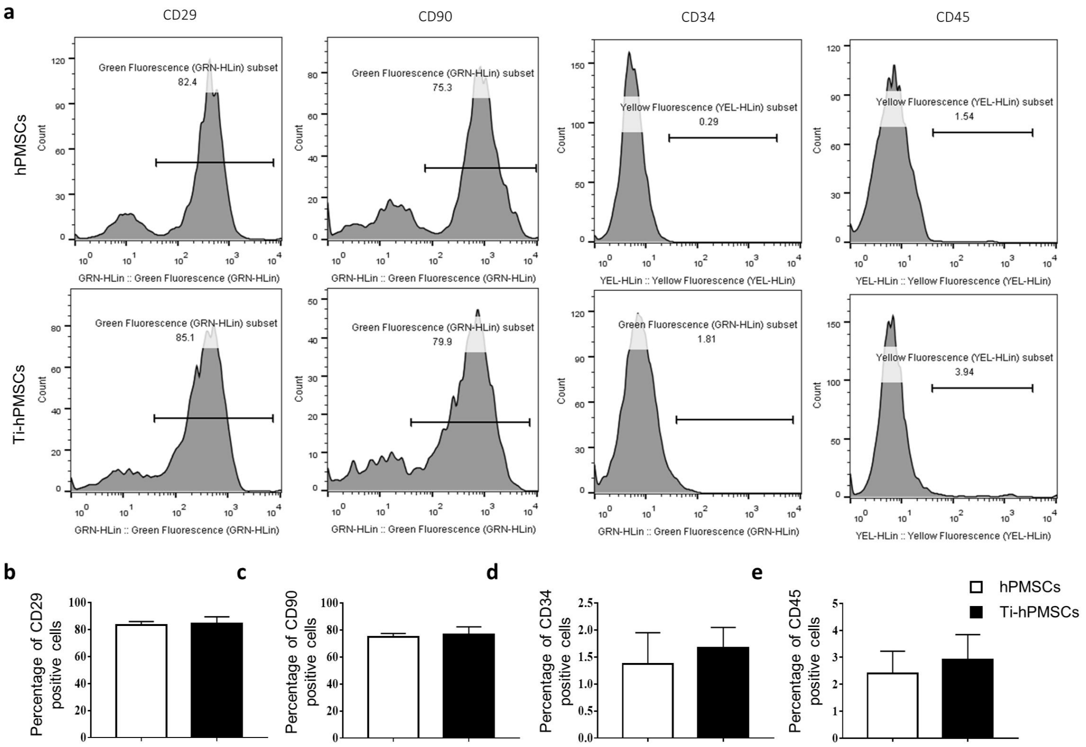

Supplementary Figure 1. Effects of Ti particles on surface marker expression of hPMSCs. (a) The representative expression of surface markers CD29, CD90, CD34 and CD45 by FCA. **(b-e)** Quantitative analysis of CD29, CD90, CD34 and CD45 expression. All the data are presented as the average ± standard deviation (S.D.).

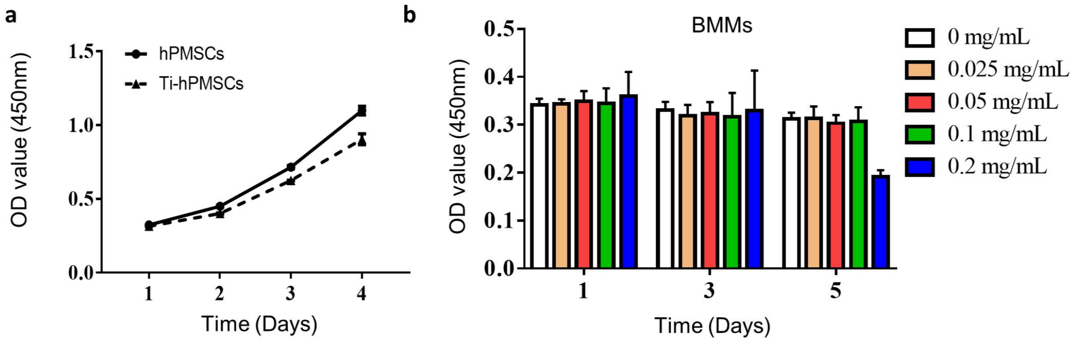

Supplementary Figure 2. Effect of Ti particles on the proliferation of hPMSCs and BMMs. (a) CCK-8 analysis of hPMSCs pretreated with or without Ti particles. **(b)** CCK-8 analysis of BMMs cultured with different concentrations of Ti particles, n=3. All the data are presented as the average ± standard deviation (S.D.).

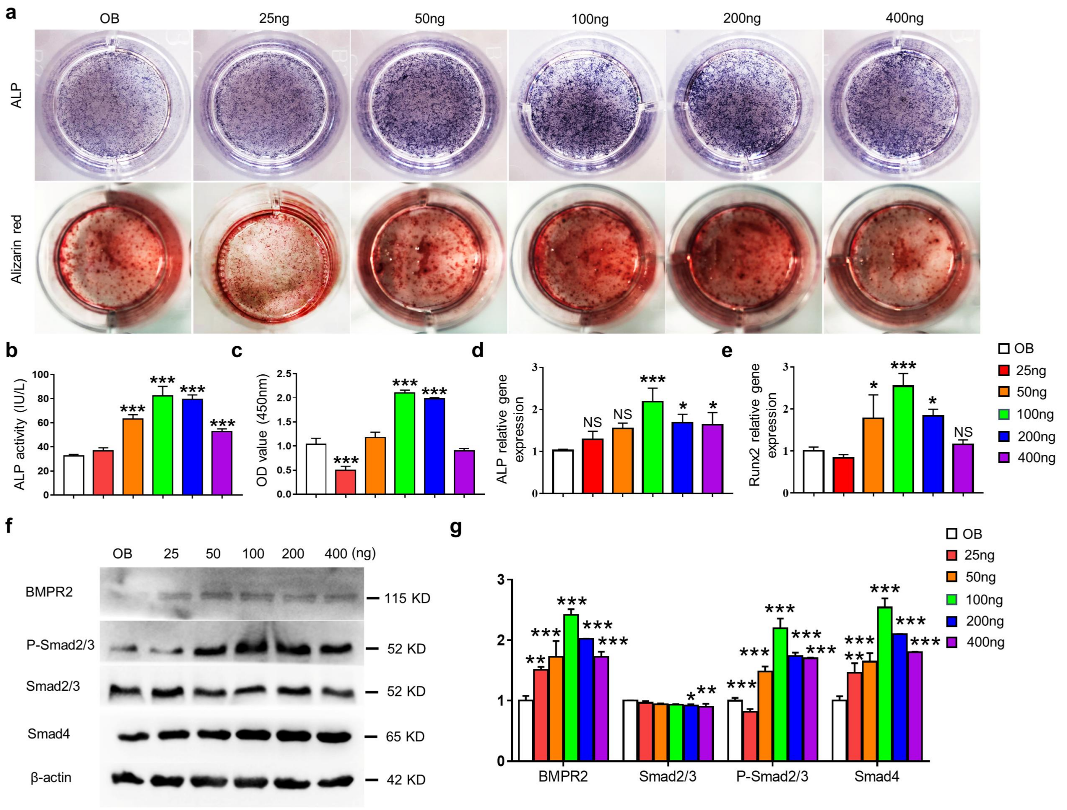

Supplementary Figure 3. Osteogenesis of hPMSCs in the presence of different concentrations of BMP2pp. hPMSCs were osteo-induced with BMP2pp (25, 50, 100, 200 and 400 ng/mL). **(a)** Representative gross images of ALP (7 days) and alizarin red S staining (14 days) of hPMSC-derived osteoblasts. **(b-c)** Quantitative analysis of ALP activity and ARS staining. **(d-e)** ALP and Runx2 gene expression of hPMSC-derived osteoblasts after 7 days of culture by qRT – PCR. **(f)** BMPR2, Smad2/3, P-Smad2/3 and Smad4 protein expression of hPMSC-derived osteoblasts after 5 days of culture by western blot analysis. **(g)** The relative grey level of corresponding to BMPR2, Smad2/3, P-Smad2/3 and Smad4 were quantified by using ImageJ. NS means no significance compared with the OB group, n=3. All the data are presented as the average ± standard deviation (S.D.).

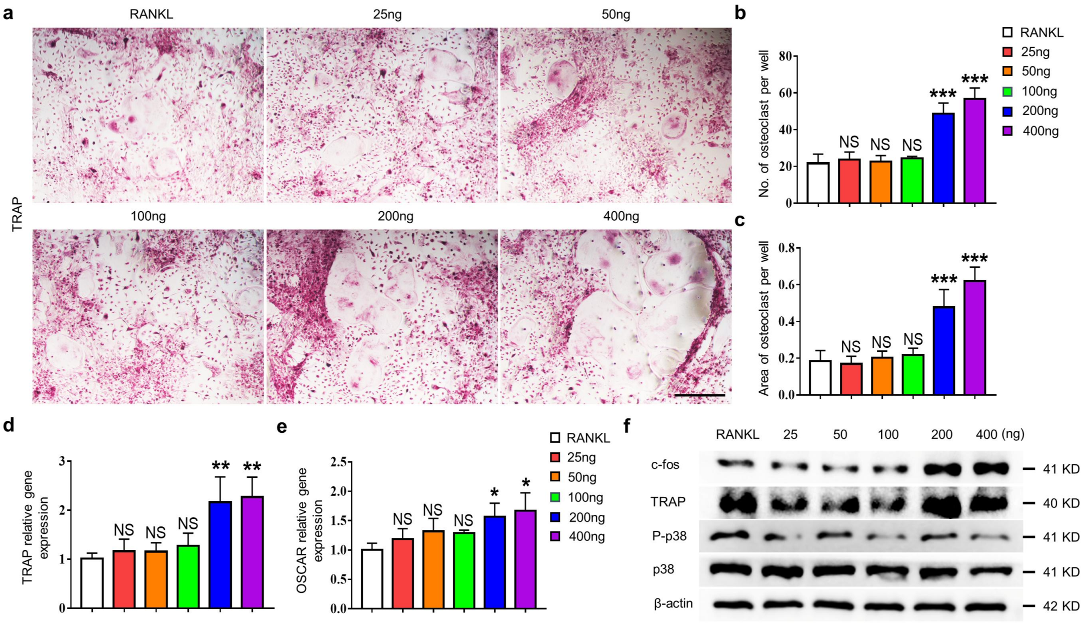

Supplementary Figure 4. Osteoclastogenesis of mice BMMs in the presence of different concentrations of BMP2pp. Mice BMMs were induced into osteoclasts with BMP2pp (25, 50, 100, 200 and 400 ng/mL) for 5 days. **(a)** Representative images of TRAP staining. Scale bar: 200 μm. **(b)** Number of osteoclasts per well. **(c)** Area of osteoclasts per well. **(d-e)** TRAP and OSCAR gene expression of BMMs by qRT−PCR. **(f)** C-fos, TRAP, p38 and P-p38 protein expression of BMMs by western blot analysis. NS means no significance compared with the RANKL group, n=3. All the data are presented as the average ± standard deviation (S.D.).

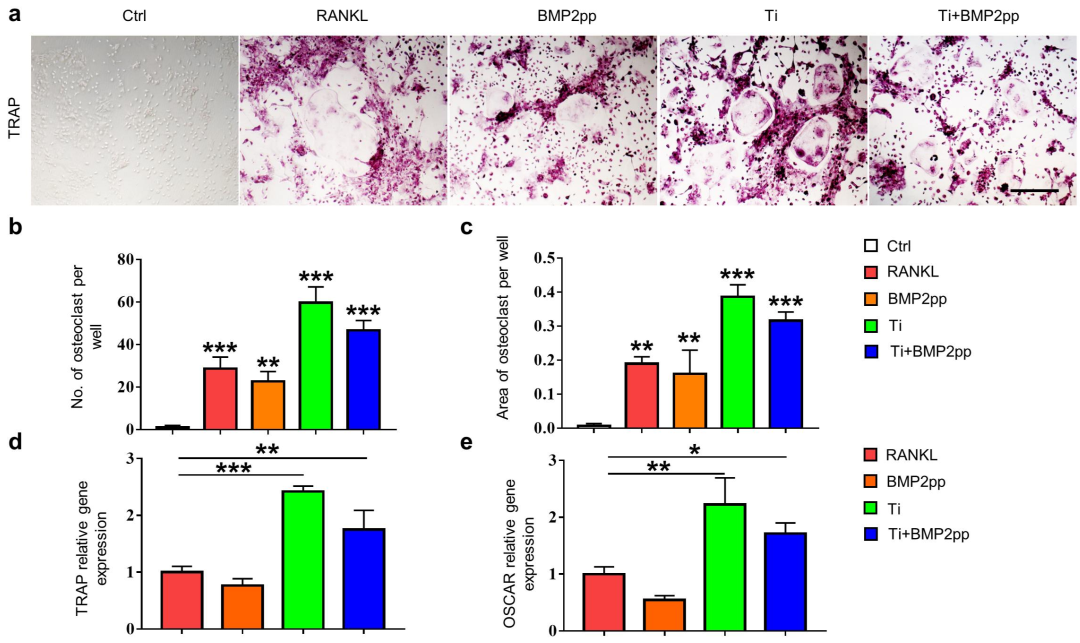

Supplementary Figure 5. Effects of BMP2pp on osteoclastic differentiation of BMMs induced by Ti particles. BMMs treated with Ti particles were induce into osteoclasts for 5 days with or without BMP2pp. **(a)** Representative images of TRAP staining. Scale bar: 200 μm. **(b)** Number of osteoclasts per well. **(c)** Area of osteoclasts per well. **(d-e)** TRAP and OSCAR gene expression by qRT–PCR. n=3. All the data are presented as the average ± standard deviation (S.D.).

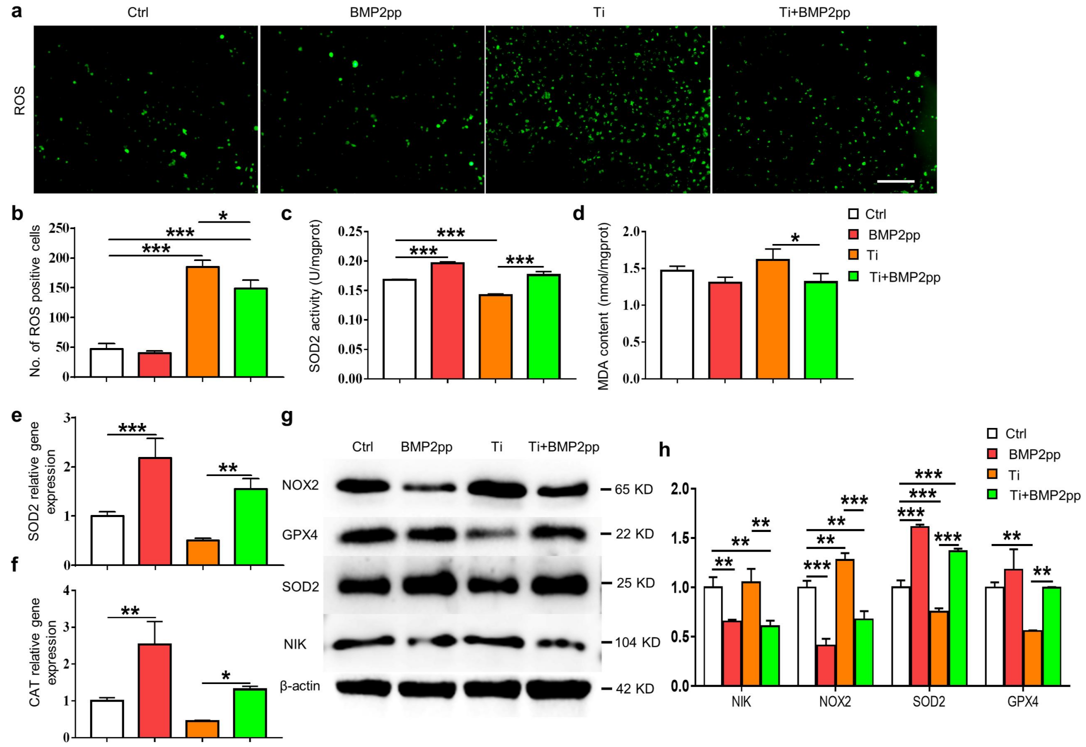

Supplementary Figure 6. Effects of BMP2pp on oxidative stress level of BMMs induced by Ti particles. BMMs treated with Ti particles were cultured with or without BMP2pp for 24 hrs. **(a)** Representative images of ROS positive (green) BMMs. Scale bar: 100 μm. **(b)** Number of ROS positive BMMs. **(c)** SOD2 activity of BMMs. **(d)** MDA content of BMMs. **(e-f)** SOD2 and CAT (catalase) gene expression in BMMs by qRT−PCR. **(g)** NOX2, GPX4, SOD2 and NIK protein expression in BMMs by western blot analysis. n=3. All the data are presented as the average ± standard deviation (S.D.).

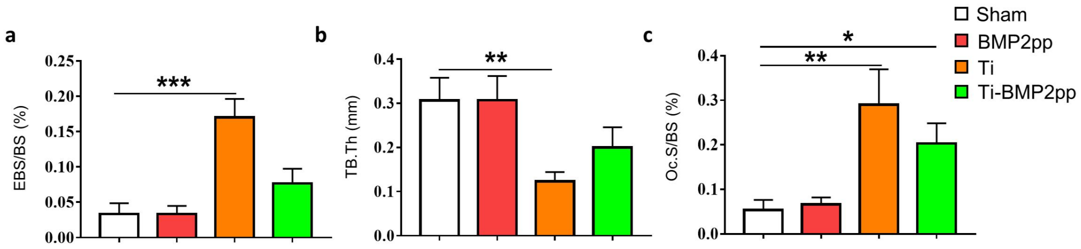

Supplementary Figure 7. BMP2pp alleviated Ti induced calvarial bone osteolysis *in vivo*. (a-c) The ratio of eroded bone surface to total bone surface (EBS/BS, %), trabecular bone thickness and the ratio of osteoclast surface to total bone surface (Oc.S/BS, %) of mouse calvarial bone in different groups by μ-CT 3D reconstruction analysis. n=8. All the data are presented as the average ± standard deviation (S.D.).

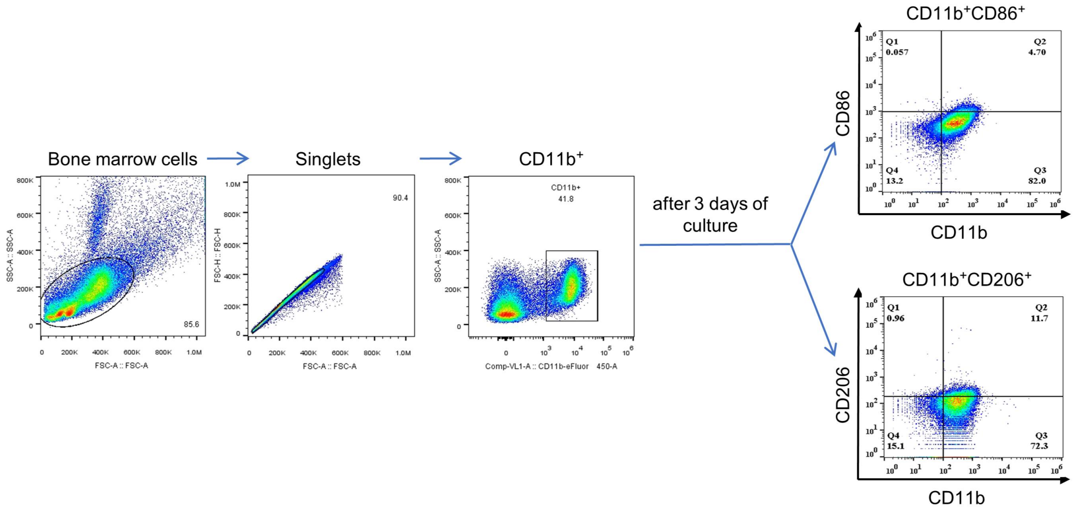

Supplementary Figure 8. Flow cytometry gating strategy. Total mice bone marrow cells were first gated on a forward scatter (FS)/side scatter (SS) plot to get the single cell, and then gated on the $CD11b^+$ population. These cells were further gated for the subsets of interest, $CD11b^+CD86^+$ cells or $CD11b^+CD206^+$ cells after 3 days of culture.

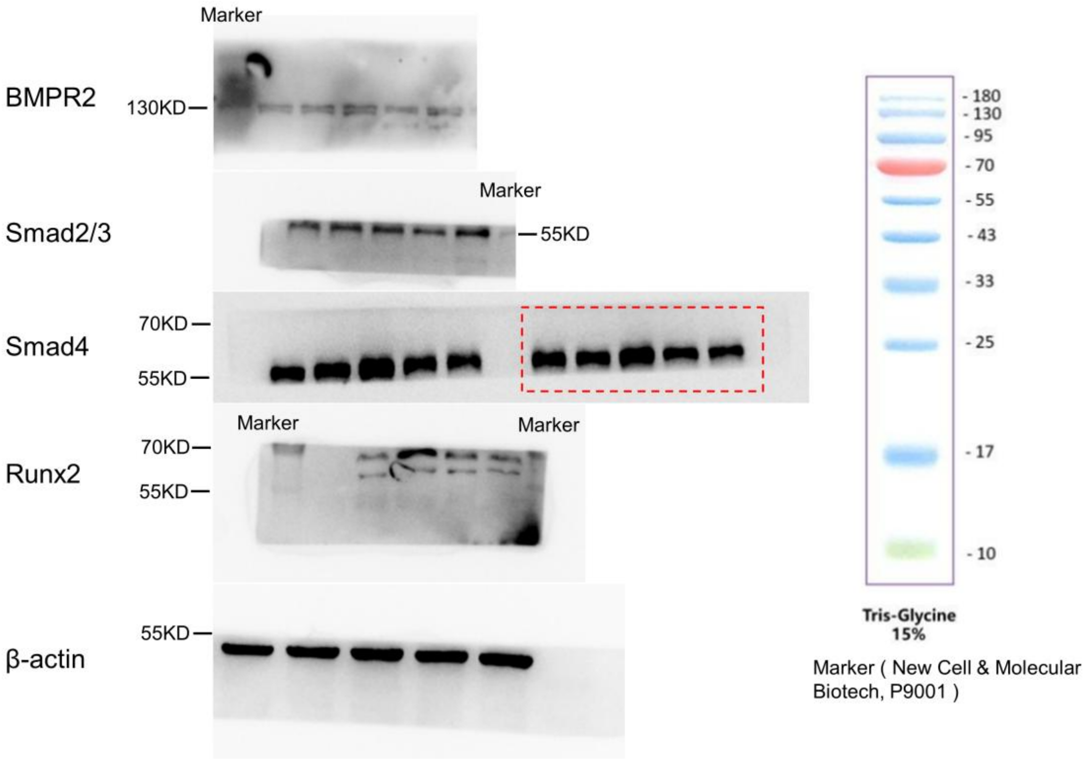

Supplementary Figure 9. Raw images of western blots in Figure 1h and the marker information.

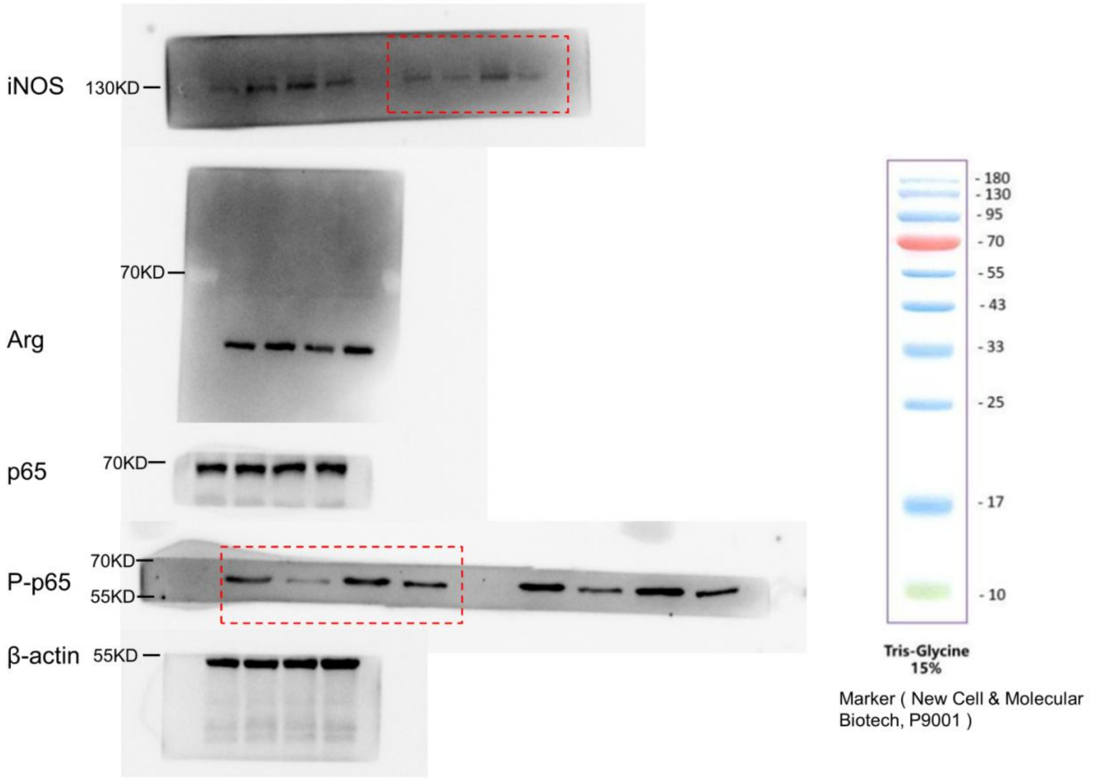

Supplementary Figure 10. Raw images of western blots in Figure 2k and the marker information.

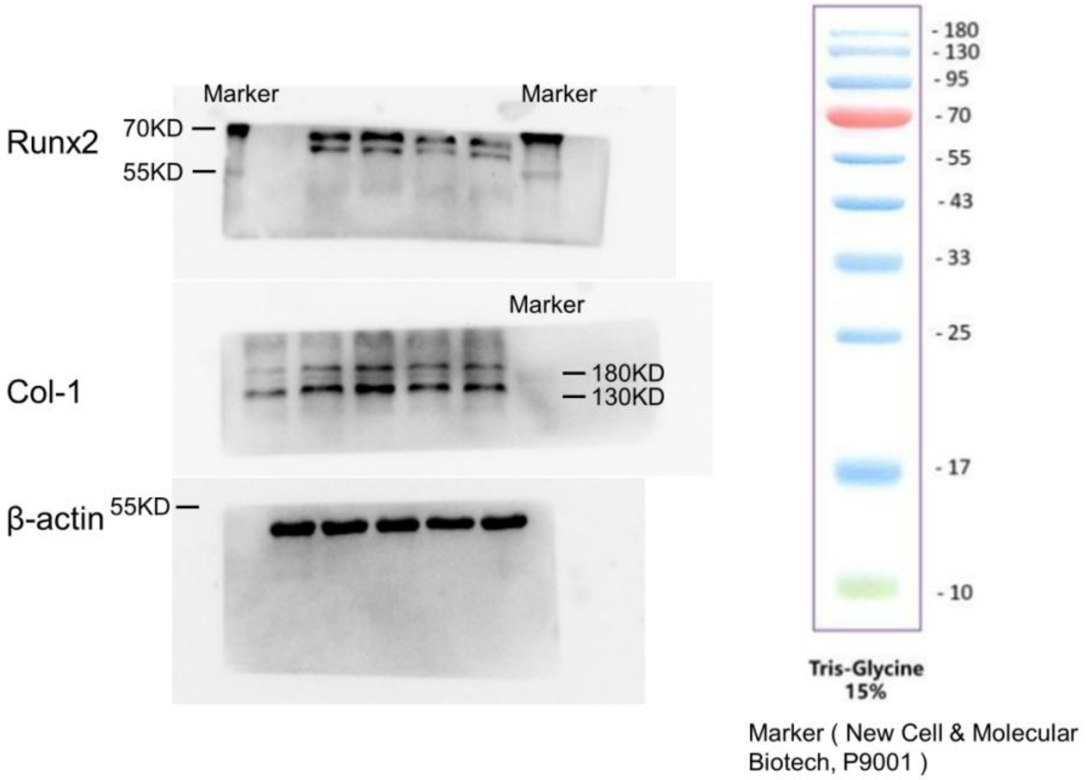

Supplementary Figure 11. Raw images of western blots in Figure 3g and the marker information.

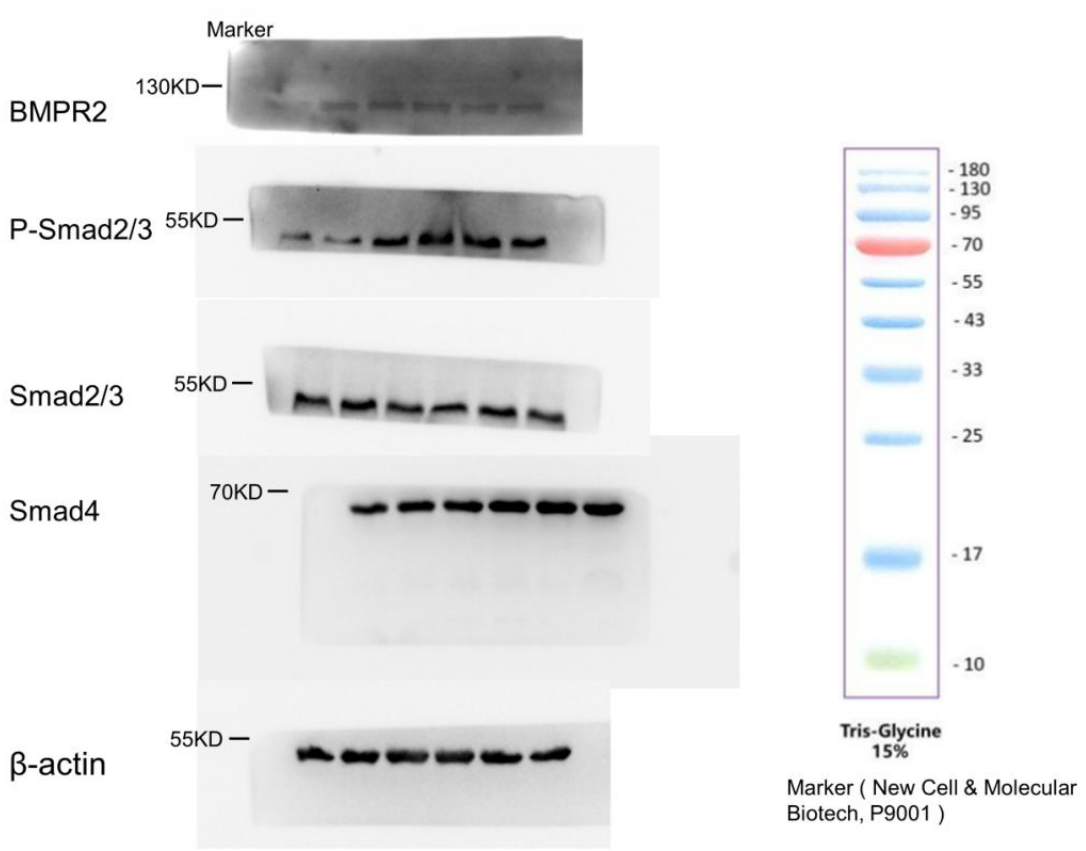

Supplementary Figure 12. Raw images of western blots in Supplementary Figure 3f and the marker information.

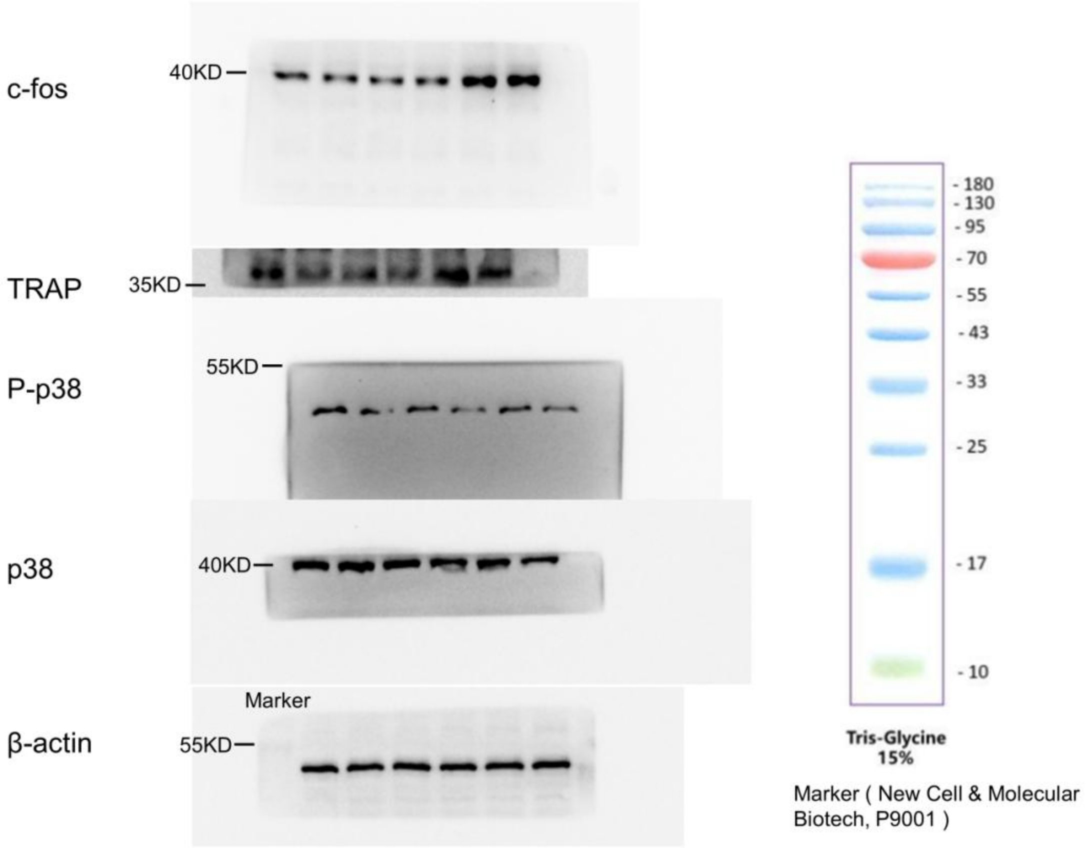

Supplementary Figure 13. Raw images of western blots in Supplementary Figure 4f and the marker information.

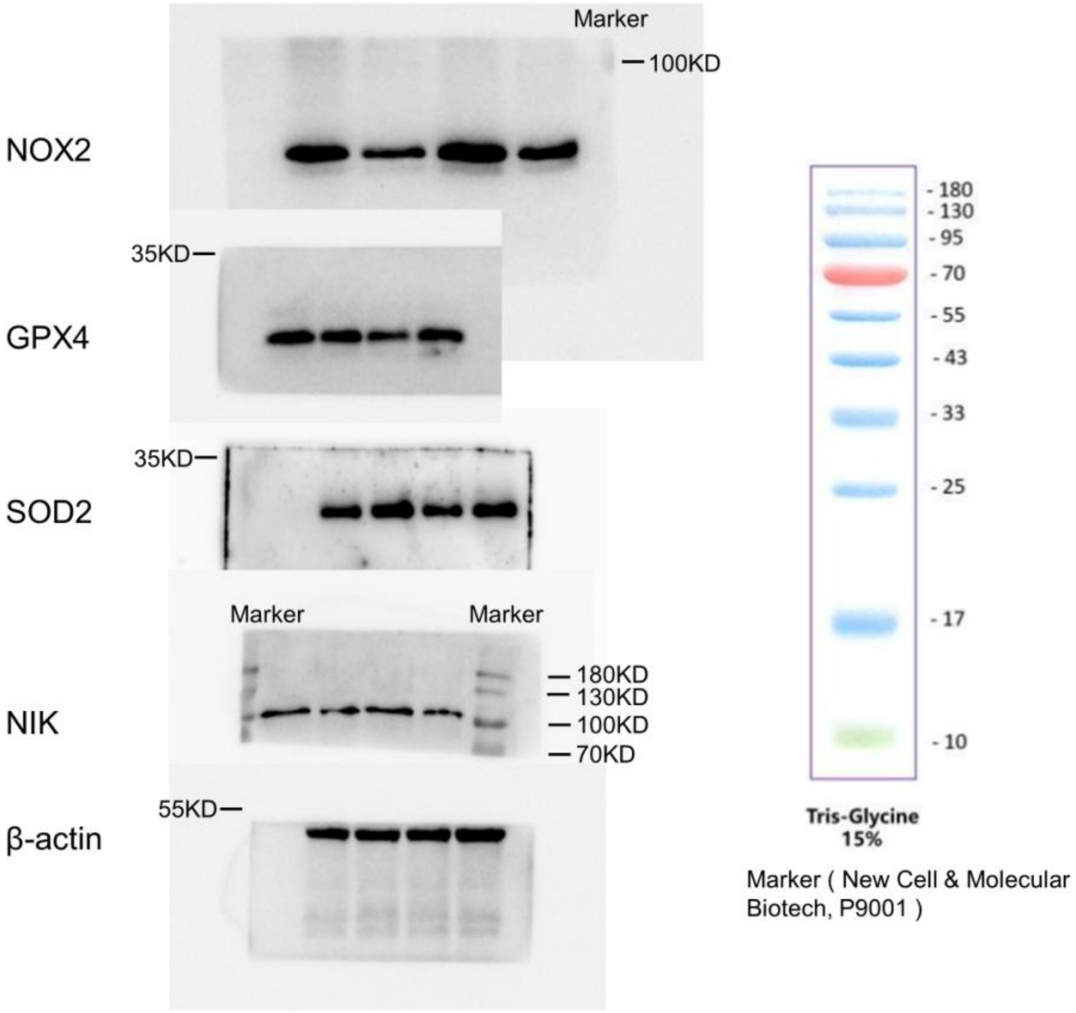

Supplementary Figure 14. Raw images of western blots in Supplementary Figure 6g and the marker information.

Supplementary Table 1. Primers of human genes for qRT–PCR

Genes	F Sequences (5'— 3')	R Sequences (5'— 3')
GAPDH	AGAAAAACCTGCCAAATATGATGAC	TGGGTGTCGCTGTTGAAGTC
ALP	AGCACTCCCACTTCATCTGGAA	GAGACCCAATAGGTAGTCCAC ATTG
RUNX2	AGAAGGCACAGACAGAAGCTTGA	AGGAATGCGCCCTAAATCACT
COL-1	CAGCCGCTTCACCTACAGC	TTTTGTATTCAATCACTGTCTT GCC

Supplementary Table 2. Primers of mice genes for qRT–PCR

Genes	F Sequences (5'— 3')	R Sequences (5'— 3')
GAPDH	ACCCAGAAGACTGTGGATGG	CACATTGGGGGTAGGAACAC
TRAP	CTGGAGTGCACGATGCCAGCGACA	TCCGTGCTCGGCGATGGACCAGA
Oscar	CTGCTGGTAACGGATCAGCTCCCC AGA	CCAAGGAGCCAGAACCTTCGAAA CT
TNF-α	TGTTACTGCCAGGACCCATA	CTTCCTTGATGGTCTCCACA
iNOS	ATAGTCCTTCCTACCCCAATTTCC	GATGAATTGGATGGTCTTGGTCC
IL-1β	AGTTGACGGACCCCAAAAGA	GGACAGCCCAGGTCAAAGG
TNF-β	CAGTACAGCAAGGTCCTTGC	ACGTAGTAGACGATGGGCAG
IL-10	GCTCTTACTGACTGGCATGAG	CGCAGCTCTAGGAGCATGTG
Arg	GGTCTCAACCCCCAGCTAGT	GCCGATGATCTCTCTCAAGTGAT
SOD2	GGGGATTGATGTGTGGGAGCACG	AGACAGGACGTTATCTTGCTG GGA
CAT	TGGGATCTCGTTGGAAATAACAC	TCAGGACGTAGGCTCCAGAAG
