# Supplementary material for: BMP-2 functional polypeptides relieve osteolysis via bi-regulating bone formation and resorption coupled with macrophage polarization
Source: NPJ Regen Med. 2023 Feb 9;8:6. doi: 10.1038/s41536-023-00279-2 (PMC9911742; doi:10.1038/s41536-023-00279-2)
Supplement: Supplementary file 2 — Reporting Summary [file 41536_2023_279_MOESM2_ESM.pdf]

nature portfolio

Corresponding author(s): DBPR NPJREGENMED-01075R2

Last updated by author(s): Jan 11, 2023

Reporting Summary

Nature Portfolio wishes to improve the reproducibility of the work that we publish. This form provides structure for consistency and transparency in reporting. For further information on Nature Portfolio policies, see our Editorial Policies and the Editorial Policy Checklist.

Statistics

For all statistical analyses, confirm that the following items are present in the figure legend, table legend, main text, or Methods section.

n/a	Confirmed	
☐	☒	The exact sample size (*n*) for each experimental group/condition, given as a discrete number and unit of measurement
☐	☒	A statement on whether measurements were taken from distinct samples or whether the same sample was measured repeatedly
☐	☒	The statistical test(s) used AND whether they are one- or two-sided<br>*Only common tests should be described solely by name; describe more complex techniques in the Methods section.*
☐	☒	A description of all covariates tested
☐	☒	A description of any assumptions or corrections, such as tests of normality and adjustment for multiple comparisons
☐	☒	A full description of the statistical parameters including central tendency (e.g. means) or other basic estimates (e.g. regression coefficient) AND variation (e.g. standard deviation) or associated estimates of uncertainty (e.g. confidence intervals)
☒	☐	For null hypothesis testing, the test statistic (e.g. *F*, *t*, *r*) with confidence intervals, effect sizes, degrees of freedom and *P* value noted<br>*Give P values as exact values whenever suitable.*
☒	☐	For Bayesian analysis, information on the choice of priors and Markov chain Monte Carlo settings
☒	☐	For hierarchical and complex designs, identification of the appropriate level for tests and full reporting of outcomes
☒	☐	Estimates of effect sizes (e.g. Cohen's *d*, Pearson's *r*), indicating how they were calculated

Our web collection on statistics for biologists contains articles on many of the points above.

Software and code

Policy information about availability of computer code

Data collection	SkyScan1176 (1.20.3.0), ZEN (blue edition)2.3, Attune NxT Software v3.2.1, Quantity One (4.6.9)
Data analysis	CT Analyser (1.20.3.0), Flowjo v10, Quantity One (4.6.9), GraphPad Prism (9.4.1)

For manuscripts utilizing custom algorithms or software that are central to the research but not yet described in published literature, software must be made available to editors and reviewers. We strongly encourage code deposition in a community repository (e.g. GitHub). See the Nature Portfolio guidelines for submitting code & software for further information.

Data

Policy information about availability of data

All manuscripts must include a data availability statement. This statement should provide the following information, where applicable:

- Accession codes, unique identifiers, or web links for publicly available datasets
- A description of any restrictions on data availability
- For clinical datasets or third party data, please ensure that the statement adheres to our policy

The data used to support the findings of this study are available from the corresponding author on request.

Human research participants

Policy information about studies involving human research participants and Sex and Gender in Research.

Reporting on sex and gender	*Use the terms sex (biological attribute) and gender (shaped by social and cultural circumstances) carefully in order to avoid confusing both terms. Indicate if findings apply to only one sex or gender; describe whether sex and gender were considered in study design whether sex and/or gender was determined based on self-reporting or assigned and methods used. Provide in the source data disaggregated sex and gender data where this information has been collected, and consent has been obtained for sharing of individual-level data; provide overall numbers in this Reporting Summary. Please state if this information has not been collected. Report sex- and gender-based analyses where performed, justify reasons for lack of sex- and gender-based analysis.*
Population characteristics	*Describe the covariate-relevant population characteristics of the human research participants (e.g. age, genotypic information, past and current diagnosis and treatment categories). If you filled out the behavioural & social sciences study design questions and have nothing to add here, write "See above."*
Recruitment	*Describe how participants were recruited. Outline any potential self-selection bias or other biases that may be present and how these are likely to impact results.*
Ethics oversight	*Identify the organization(s) that approved the study protocol.*

Note that full information on the approval of the study protocol must also be provided in the manuscript.

Field-specific reporting

Please select the one below that is the best fit for your research. If you are not sure, read the appropriate sections before making your selection.

☒ Life sciences ☐ Behavioural & social sciences ☐ Ecological, evolutionary & environmental sciences

For a reference copy of the document with all sections, see nature.com/documents/nr-reporting-summary-flat.pdf

Life sciences study design

All studies must disclose on these points even when the disclosure is negative.

Sample size	No sample size calculation was performed
Data exclusions	No data were excluded for the analyses.
Replication	All experiments were repeated at least three times, and the data were obtained at least in triplicate. All attempts at replication were successful.
Randomization	7-week-old male C57BL/6 mice were randomly divided into 4 groups (n=8/group).
Blinding	The investigators were not blinded to group allocation during data collection and analysis, because the molecular biology methods were used to further analysis the differences between different groups, including immunohistochemistry staining, qRT-PCR and western blot.

Reporting for specific materials, systems and methods

We require information from authors about some types of materials, experimental systems and methods used in many studies. Here, indicate whether each material, system or method listed is relevant to your study. If you are not sure if a list item applies to your research, read the appropriate section before selecting a response.

Materials & experimental systems

n/a	Involved in the study	
☐	☒	Antibodies
☒	☐	Eukaryotic cell lines
☒	☐	Palaeontology and archaeology
☐	☒	Animals and other organisms
☒	☐	Clinical data
☒	☐	Dual use research of concern

Methods

n/a	Involved in the study	
☒	☐	ChIP-seq
☐	☒	Flow cytometry
☒	☐	MRI-based neuroimaging

Antibodies

Antibodies used	All the western blot primary antibodies were from Abcam, including Runt-related transcription factor 2 (Runx2, ab236639), NF-κB-inducing kinase (NIK, ab203568), p65 (ab32536), P-p65 (ab76302), BMP receptor type 2 (BMPR2, ab130206), Smad2/3 (ab202445), P-smad2/3 (ab254407), Smad4 (ab230815), inducible nitric oxide synthase (iNOS, ab283655), arginine (Arg, ab203490), type I collagen (Col-I, ab138492), c-fos (ab222699), TRAP (ab52750), p38 (ab170099), P-p38 (ab4822), NADPH oxidase 2 (NOX2, ab129068)， glutathione peroxidase (GPX4, ab125066), SOD2 (ab68155) and β-actin (ab8226). Antibodies for FACS were from eBioscience, including FITC-CD29 (11-0299-42), PerCP/Cy5.5-CD90 (45-0909-42), PE-CD34 (12-0349-42), PE-CD45 (12-0459-42)， APC-CD11b (17-0112-82), PE-CD86 (12-0862-82), PE-CD206 (12-2061-82) and isotype control antibody.
Validation	Data provided in the manuscript.

Animals and other research organisms

Policy information about studies involving animals; ARRIVE guidelines recommended for reporting animal research, and Sex and Gender in Research

Laboratory animals	7-week-old male C57BL/6 mice
Wild animals	The study did not involve wild animals.
Reporting on sex	The study only use male mice for in vivo experiments.
Field-collected samples	The study did not involve samples collected from the field.
Ethics oversight	All animal experimental protocols were approved by the Animal Care and Use Committee of Soochow University.

Note that full information on the approval of the study protocol must also be provided in the manuscript.

Flow Cytometry

Plots

Confirm that:

☒ The axis labels state the marker and fluorochrome used (e.g. CD4-FITC).

☒ The axis scales are clearly visible. Include numbers along axes only for bottom left plot of group (a 'group' is an analysis of identical markers).

☒ All plots are contour plots with outliers or pseudocolor plots.

☒ A numerical value for number of cells or percentage (with statistics) is provided.

Methodology

Sample preparation	hPMSCs were isolated from healthy donor and cultured as described previously. hPMSCs were treated with or without Ti particles (0.1 mg/mL) for 5 days. The cells were collected and stained with anti-human antibodies (eBioscience, FITC-CD29 (11-0299-42), PerCP/Cy5.5-CD90 (45-0909-42), PE-CD34 (12-0349-42), PE-CD45 (12-0459-42)) or isotype control antibody. Bone-marrow-derived macrophages (BMMs) were extracted from femurs and tibiae bone marrow cells of 6-week-old C57/BL6 mice and cultured as described previously. BMMs (100000/well, 6-well plates) were treated with 0.1 mg/mL Ti particles and cultured with or without 100 ng/mL BMP2pp. The cells were harvested after 3 days of culture and then incubated with anti-mouse antibodies (eBioscience, APC-CD11b (17-0112-82), PE-CD86 (12-0862-82), PE-CD206 (12-2061-82)) or isotype control antibody.
Instrument	Attune NxT Acoustic Focusing Cytometer
Software	Attune NxT Software 3.2.1
Cell population abundance	Almost 80% of the cultured hPMSCs expressed the mesenchymal markers CD29 and CD90 and did not express the hematopoietic cell surface markers CD34 and CD45. Almost 78% of the cultured BMMs expressed the macrophages marker CD11b.
Gating strategy	First draw a loose gate around cell type of interest from the FSC-A x SSC-A plot and name as bone marrow cells. Then, do the single cell gating from the FSC-A x FSC-H plot and name as singlets. Use the isotype control antibodies as negative control, gate the CD11b+ cells by using APC-CD11b antibody. Finally, gate the CD11b+CD86+ cells or CD11b+CD206+ cells by using APC-CD11b, PE-CD86, PE-CD206 antibodies after 3 days of culture.

☒ Tick this box to confirm that a figure exemplifying the gating strategy is provided in the Supplementary Information.
